# CD4^+^ and CD8^+^ T cells are required to prevent SARS-CoV-2 persistence in the nasal compartment

**DOI:** 10.1126/sciadv.adp2636

**Published:** 2024-08-23

**Authors:** Meenakshi Kar, Katherine E. E. Johnson, Abigail Vanderheiden, Elizabeth J. Elrod, Katharine Floyd, Elizabeth Geerling, E. Taylor Stone, Eduardo Salinas, Stephanie Banakis, Wei Wang, Shruti Sathish, Swathi Shrihari, Meredith E. Davis-Gardner, Jacob Kohlmeier, Amelia Pinto, Robyn Klein, Arash Grakoui, Elodie Ghedin, Mehul S. Suthar

**Affiliations:** ^1^ Center for Childhood Infections and Vaccines of Children’s Healthcare of Atlanta, Department of Pediatrics, Emory University School of Medicine, Atlanta, GA, USA.; ^2^ Emory Vaccine Center, Emory University, Atlanta, GA, USA.; ^3^ Emory National Primate Research Center, Atlanta, GA, USA.; ^4^ Systems Genomics Section, Laboratory of Parasitic Diseases, DIR, NIAID, NIH, Bethesda, MD, USA.; ^5^ Center for Neuroimmunology and Neuroinfectious Diseases, Washington University School of Medicine, St. Louis, MO, USA.; ^6^ Department of Medicine, Washington University School of Medicine, St. Louis, MO, USA.; ^7^ Department of Medicine, Emory University School of Medicine, Emory University, Atlanta, GA, USA.; ^8^ Department of Molecular Microbiology and Immunology, Saint Louis University School of Medicine, Saint Louis, MO, USA.; ^9^ Department of Microbiology and Immunology, Emory University, Atlanta, GA, USA.; ^10^ Schulich School of Medicine and Dentistry, Department of Microbiology and Immunology, Western University, London, Ontario, Canada.; ^11^ Schulich School of Medicine and Dentistry, Western Institute of Neuroscience, Western University, London, Ontario, Canada.

## Abstract

SARS-CoV-2 infection induces the generation of virus-specific CD4^+^ and CD8^+^ effector and memory T cells. However, the contribution of T cells in controlling SARS-CoV-2 during infection is not well understood. Following infection of C57BL/6 mice, SARS-CoV-2–specific CD4^+^ and CD8^+^ T cells are recruited to the respiratory tract, and a vast proportion secrete the cytotoxic molecule granzyme B. Using depleting antibodies, we found that T cells within the lungs play a minimal role in viral control, and viral clearance occurs in the absence of both CD4^+^ and CD8^+^ T cells through 28 days postinfection. In the nasal compartment, depletion of both CD4^+^ and CD8^+^ T cells, but not individually, results in persistent, culturable virus replicating in the nasal epithelial layer through 28 days postinfection. Viral sequencing analysis revealed adapted mutations across the SARS-CoV-2 genome, including a large deletion in ORF6. Overall, our findings highlight the importance of T cells in controlling virus replication within the respiratory tract during SARS-CoV-2 infection.

## Teaser

CD4^+^ and CD8^+^ T cells protect against SARS-CoV-2 viral persistence within the upper respiratory tract.

## INTRODUCTION

The global impact of severe acute respiratory syndrome coronavirus 2 (SARS-CoV-2) remains devastating, with more than 773 million confirmed cases and around 7 million deaths reported worldwide by the end of December 2023 (World Health Organization COVID-19 dashboard; https://covid19.who.int). SARS-CoV-2 is primarily transmitted through respiratory droplets and targets ciliated epithelial cells in the nasal cavity, trachea, and lungs (*1*, *2*) by using the cellular receptor angiotensin-converting enzyme 2 (ACE2) (*3*–*5*). Infection of the upper respiratory tract is generally associated with a milder disease outcome, whereas dissemination to the lungs, particularly infection of the bronchi, bronchioles, and alveoli, can cause pneumonia, severe disease, acute respiratory distress syndrome, and death (*3*).

The development of mouse models of SARS-CoV-2 has enabled the study of transmission, immunity, and pathogenesis of this virus (*6*–*10*). The ancestral SARS-CoV-2 strain does not replicate in conventional laboratory mice due to inefficient spike protein binding to the murine ACE2 (*11*, *12*). To overcome this limitation, several mouse models have been developed, including human ACE2 (hACE2) transgenic mice that express hACE2 transiently after transduction of hACE2 with viral vectors (e.g., adenovirus) (*13*, *14*), K18-hACE2 transgenic mice (*15*), or the use of mouse-adapted strains (*7*, *16*, *17*). A naturally occurring spike mutation at position N501Y, which is found in many SARS-CoV-2 variants (Alpha, Beta, Gamma, and Mu variants), increases the affinity of SARS-CoV-2 spike protein for the murine ACE2 receptor and allows for infection of inbred mice (*11*, *18*–*21*). Infection of conventional laboratory mice with naturally occurring N501Y spike mutations shows inflammatory infiltrates, alveolar edema, and alveolitis (*13*, *16*, *22*, *23*). Using this model, we recently identified that the CCR2-monocyte signaling axis is important for controlling virus replication and dissemination within the lungs and protection against the SARS-CoV-2 B.1.351 (“Beta”) variant (*22*).

Following SARS-CoV-2 infection, SARS-CoV-2–specific CD4^+^ and CD8^+^ T cells are detectable within the peripheral blood of patients with COVID-19 (*24*–*27*). These circulating virus-specific CD4^+^ and CD8^+^ T cells target several SARS-CoV-2 proteins, including spike and nucleocapsid, and are polyfunctional and durable with estimated half-lives of more than 200 days (*24*). SARS-CoV-2–specific CD4^+^ T cells are important in promoting antibody responses and mitigating disease severity as reduced responses associate with increased disease severity (*28*–*31*). CD8^+^ T cells appear to play a protective role with reduced responses correlating with adverse prognoses (*32*). Rapid type I interferon (IFN) responses and virus-specific CD8^+^ T cell responses coincide with milder SARS-CoV-2 infections, preceding the development of antibodies by 1 to 2 weeks (*33*). However, the role of CD4^+^ and CD8^+^ T cells within the respiratory tract is not well understood and is only beginning to be studied in the context of SARS-CoV-2 infection in humans (*34*, *35*).

In this study, we investigated the role of CD4^+^ and CD8^+^ T cells on SARS-CoV-2 infection in both the upper respiratory tract (i.e., nasal airways) and the lower respiratory tract. Following infection, the respiratory tract recruits SARS-CoV-2–specific CD4^+^ and CD8^+^ T cells, a substantial proportion of which release the cytotoxic substance granzyme B (GzB). Through T cell depletion using antibodies before infection, we discovered that CD4^+^ and CD8^+^ T cells have distinct roles in the upper and lower respiratory tract. In the lungs, T cells have a limited impact on viral control, as viral clearance occurs even in the absence of both CD4^+^ and CD8^+^ T cells up to 28 days postinfection (p.i.). Conversely, in the nasal compartment, depleting both CD4^+^ and CD8^+^ T cells (but not individually) leads to persistent and culturable virus replication in the nasal compartment for 28 days p.i. Using in situ hybridization (ISH), we observed persistent SARS-CoV-2 infection in the nasal epithelial layer of mice depleted of both CD4^+^ and CD8^+^ T cells. Sequence analysis of virus isolates from persistently infected mice revealed mutations spanning the entire genome. In summary, our findings underscore the crucial role of T cells in controlling virus replication within the respiratory tract during SARS-CoV-2 infection.

## RESULTS

### SARS-CoV-2 variant B.1.351 replicates in the lungs and nasal airways of C57BL/6 mice

**Fig. 1.**
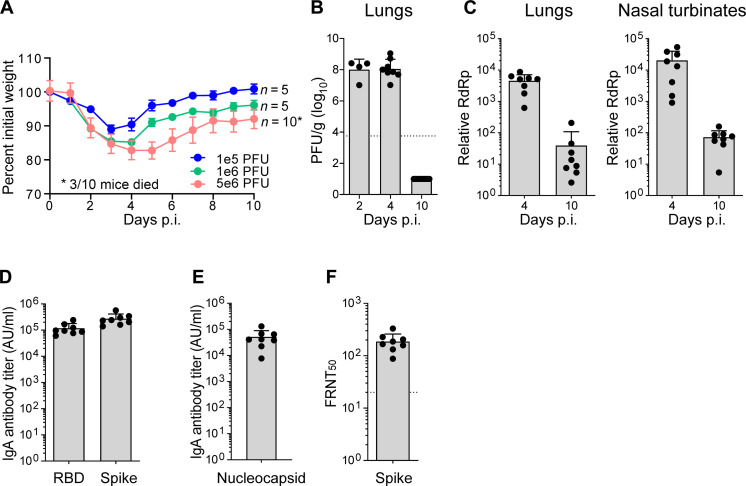
SARS-CoV-2 variant B.1.351 replicates in the lungs and nasal airways of C57BL/6 mice. C57BL/6 mice were infected with the SARS-CoV-2 B.1.351 (Beta) variant or an equal volume of saline for mock mice. (**A**) Percent of initial weight for Beta infected mice at indicated PFUs over 10 days. (**B**) Quantification of infectious virus at indicated days p.i. as measured by plaque assay and expressed as PFU per gram of lung tissue. (**C**) Quantification of viral RNA by qRT-PCR for SARS-CoV-2 RdRp in the lungs (left) and nasal turbinates (right). CT values represented as relative fold change over mock (log_10_). IgG antibody titers against (**D**) RBD and spike and (**E**) nucleocapsid as measured by an electrochemiluminescent multiplex immunoassay and reported as arbitrary units per ml (AU/ml) and normalized by a standard curve for the B.1.351 SARS-CoV-2 variant. (**F**) Neutralizing antibody response measured as 50% inhibitory titer (FRNT_50_) by focus reduction neutralization assay. Graphs show mean ± SD. Results are representative of data from two independent experiments. Day 2 p.i. (*n* = 4), day 4 p.i. (*n* = 8), and day 10 p.i. (*n* = 10).

The emergence of SARS-CoV-2 variants encoding an N501Y mutation within the spike protein allows for productive infection of conventional laboratory mice. Previously, we and others have shown that infection of C57BL/6 mice with the B.1.351 strain results in virus replication in the lungs that corresponds with induction of innate immune responses (*22*, *36*). To further characterize this model, we infected C57BL/6 mice with doses of B.1.351 ranging from 10^5^ to 10^6^ plaque-forming units (PFU) through the intranasal route. We found that increasing virus inoculum led to increased weight loss with mice infected with 5 × 10^6^ PFU showing nearly 20% weight loss with 30% mortality (Fig. 1A). For subsequent experiments, we selected 1 × 10^6^ PFU as the viral dose as we observed consistent weight loss in the absence of mortality. We next evaluated the kinetics of virus replication in the nasal airways and lungs. Infectious virus in the lungs, as measured by plaque assay on Vero-ACE2-TMPRSS2 overexpressing cells, peaked between days 2 and 4 post-infection (pi) and reached undetectable levels by day 10 p.i. (Fig. 1B). As a more sensitive measure of virus replication, we measured viral RNA-dependent RNA polymerase (RdRp) levels by quantitative reverse transcription polymerase chain reaction (qRT-PCR) and observed 116-fold reduction in virus replication in the lungs and nasal turbinates (Fig. 1C). A hallmark of SARS-CoV-2 infection is the generation of systemic spike- and nucleocapsid-specific antibody responses (*37*). Following infection, B.1.351-infected mice generated robust immunoglobulin G (IgG) responses against the receptor binding domain (RBD) [geometric mean titer (GMT) 107839], Spike (GMT 249211), and Nucleocapsid (GMT 39803) that corresponded to live virus neutralization activity [50% focus reduction neutralization test (FRNT_50_) GMT 175] (Fig. 1, D to F).

### SARS-CoV-2 B.1.351 infection triggers antigen-specific effector T cell responses in the upper and lower respiratory tract

**Fig. 2.**
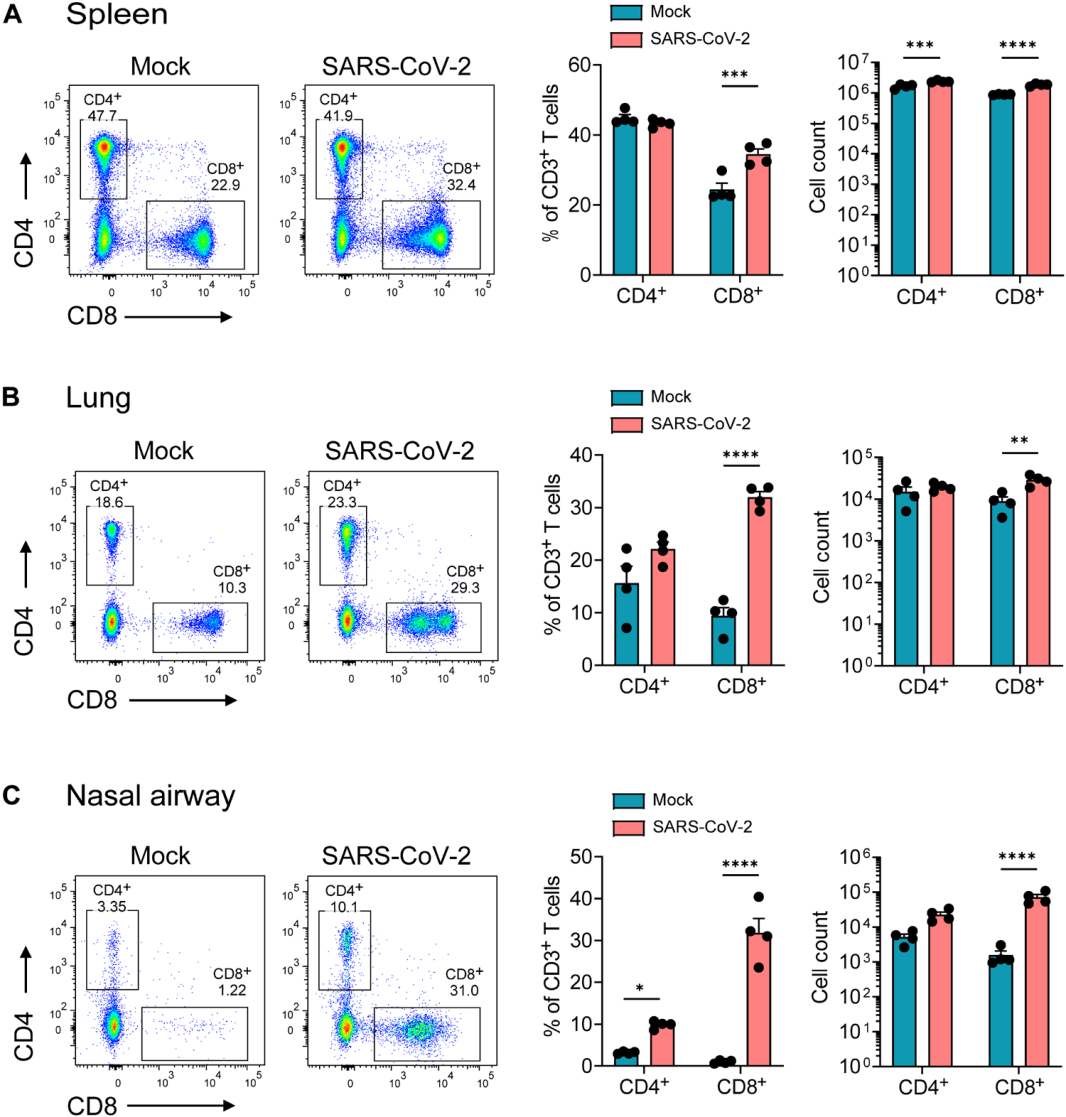
SARS-CoV-2 B.1.351 infection leads to increased infiltration of CD8^+^ T cells in the respiratory tract but not in the periphery. C57BL/6 mice were infected with the SARS-CoV-2 B.1.351 (Beta) variant at 10^6^ PFU intranasally, and at day 7 pi, spleen, lungs, and nasal airway tissues were harvested, processed for flow cytometry, and analyzed via FlowJo. Frequency and cell count for CD4^+^ and CD8^+^ T cells in (**A**) spleen, (**B**) lungs, and (**C**) nasal airway; representative flow plots on the left, frequency of cells in the middle, and cell counts on the right. Graphs show mean ± SD. A two-way analysis of variance (ANOVA) statistical test was performed. *P < 0.05, **P < 0.01, ***P < 0.001, and ****P < 0.0001; no symbol, not significant. Results are representative of data from three independent experiments with five mice per group.

Previous studies in humans have shown that SARS-CoV-2 infection can trigger antigen-specific T cell responses within the nasal compartment, lungs, and periphery (e.g., blood) (*24*, *34*, *38*). We next evaluated T cell responses within the respiratory tract and periphery of SARS-CoV-2–infected mice. Following B.1.351 infection, we isolated immune cells from the nasal compartment, lungs, and analyzed CD4^+^ and CD8^+^ T cells by flow cytometry. Before harvesting tissues, mice were intravitally labeled with CD45 antibody conjugated to phycoerythrin (PE) to allow identification of circulating (CD45-PE positive) and tissue-resident/parenchymal cells (CD45-PE negative) within the respiratory tract (gating strategy shown in fig. S1). On day 7 pi, within the spleen, we observed a modest, yet significant, increase in the frequency of CD8^+^ T (1.4-fold) cells but not CD4^+^ T cells (Fig. 2A). However, we observed a significant increase in the frequency of tissue-resident CD8^+^ T cells (3.4-fold), but not CD4^+^ T cells (1.4-fold), within the lungs (Fig. 2B), and in the nasal compartment, we observed a significant increase in CD8^+^ (32-fold) and CD4^+^ (3-fold) both the T cells (Fig. 2, B and C).

**Fig. 3.**
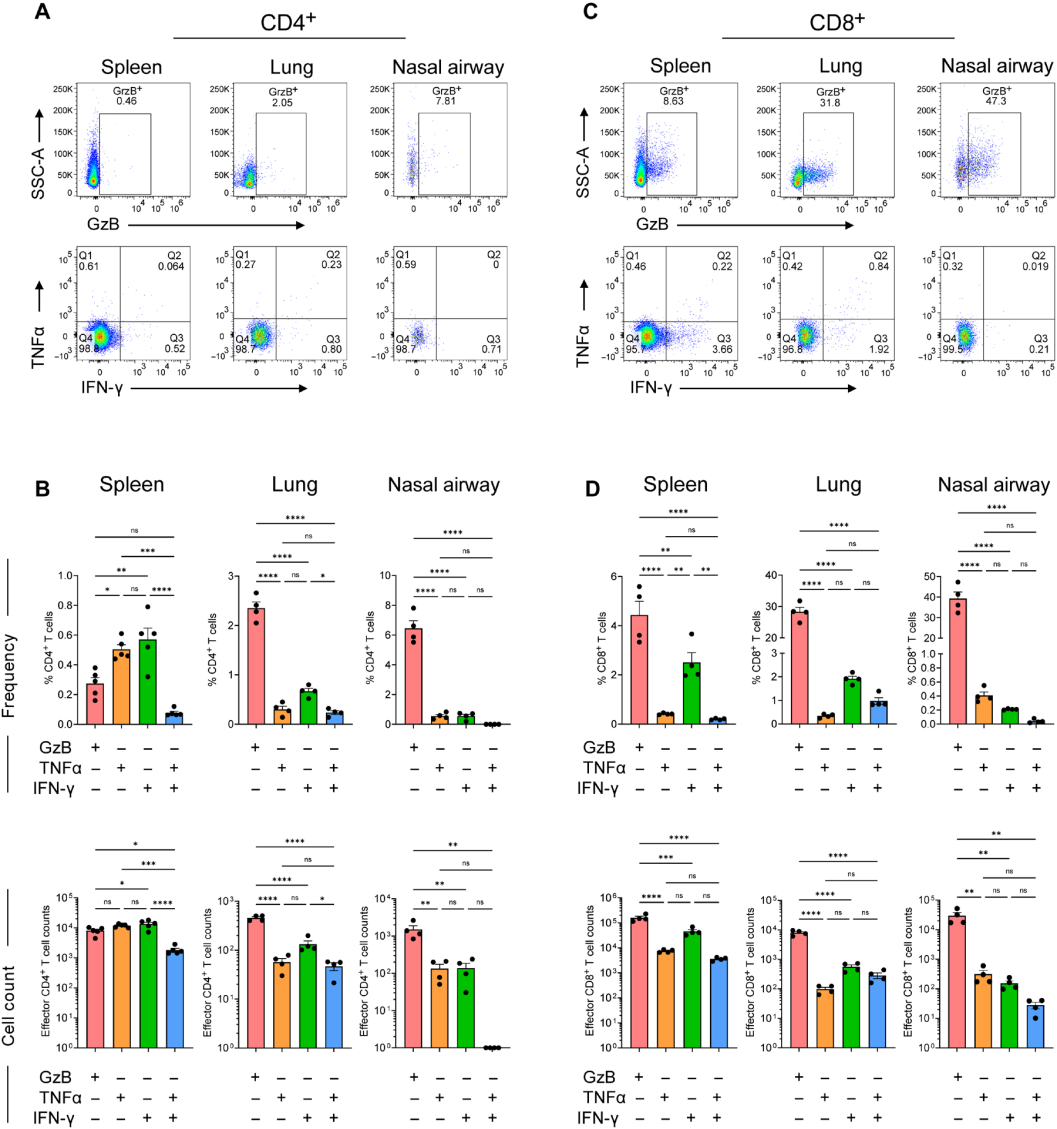
SARS-CoV-2 B.1.351 infection triggers antigen-specific effector T cell responses in the upper and lower respiratory tract. C57BL/6 mice were infected with the SARS-CoV-2 B.1.351 (Beta) variant at 10^6^ PFU intranasally, and at day 7 pi, virus-specific CD4^+^ and CD8^+^ T cell responses were evaluated by ex vivo peptide stimulation using spike peptide pools in spleen, lungs, and nasal airways. (**A**) Representative flow plots for GzB (top), TNFα, and IFN-γ (bottom) expression in CD4^+^ T cells in spleen, lungs, and nasal airways. (**B**) Frequency (top) and cell counts (bottom) of CD4^+^ T cells positive for GzB, TNFα, and IFN-γ expression in spleen, lungs, and nasal airways. (**C**) Representative flow plots for GzB (top), TNFα, and IFN-γ (bottom) expression in CD8^+^ T cells in spleen, lungs, and nasal airways. (**D**) Frequency (top) and cell counts (bottom) of CD4^+^ T cells positive for GzB, TNFα, and IFN-γ expression in spleen, lungs, and nasal airways. Graphs show mean ± SD. A two-way ANOVA statistical test was performed. *P < 0.05, **P < 0.01, ***P < 0.001, and ****P < 0.0001; no symbol, not significant (ns). Results are representative of data from three independent experiments with five mice per group.

We next evaluated the antigen-specific T cell responses by performing ex vivo peptide restimulation with a spike peptide pool followed by intracellular staining and flow cytometry analysis (Fig. 3A and fig. S2). For antigen-specific CD4^+^ T cells, in both the lungs and nasal compartment, we observed higher cell frequencies and counts of GzB secreting as compared to cytokine-secreting cells (Fig. 3B). Specifically, we observed 2.35% GzB^+^, 0.3% tumor necrosis factor–α–positive (TNFα^+^), 0.67% IFN-γ^+^, and 0.24% TNFα^+^IFN-γ^+^ CD4^+^ T cells in the lungs and 6.45% GzB^+^, 0.56% TNFα^+^, 0.56% IFN-γ^+^, and 0% TNFα^+^IFN-γ^+^ CD4^+^ T cells in the nasal compartment. In the spleen, we observed 0.27% GzB^+^, 0.50% TNFα^+^, 0.57% IFN-γ^+^, and 0.08% TNFα^+^IFN-γ^+^ CD4^+^ T cells.

For antigen-specific CD8^+^ T cells, in the lungs, nasal compartment, and spleen, we observed higher cell frequencies and counts of GzB secreting as compared to cytokine-secreting cells (Fig. 3, C and D). We observed 28.3% GzB^+^, 0.34% TNFα^+^, 1.91% IFN-γ^+^, and 0.97% TNFα^+^IFN-γ^+^ CD8^+^ T cells in the lungs, 39.25% GzB^+^, 0.40% TNFα^+^, 0.21% IFN-γ^+^, and 0.04% TNFα^+^IFN-γ^+^ CD8^+^ T cells in the nasal compartment, and 4.42% GzB^+^, 0.41% TNFα^+^, 2.50% IFN-γ^+^, and 0.20% TNFα^+^IFN-γ^+^ CD8^+^ T cells in the spleen. We compared activation markers (CD44, KLRG1, and CD69) in CD4^+^ and CD8^+^ T cells across lungs, upper respiratory tract (URT), and spleen (fig. S3). We discovered intriguing variations in activation between upper and lower respiratory areas. In the lungs, infected samples showed increased CD44 and CD69 and, to a lesser extent, KLRG1 in contrast to mock samples. This increase in CD69 in CD4^+^ and CD8^+^ T cells could explain the infiltration of these cells in the lungs due to migration as CD69 has been implicated in regulating T cell migration (*39*). However, in the nasal airways, there was no difference in CD44 and CD69 expression in CD4^+^ and CD8^+^ T cells. Unexpectedly, there was a significant rise in KLRG1 expression in CD8^+^ T cells in the URT. In the spleen, only CD44 expression increased a little in the CD8^+^ T cells, but we did not see any changes in the CD69 or KLRG1 expression in infected samples compared to mock. These data indicate that SARS-CoV-2 infection triggers antigen-specific CD4^+^ and CD8^+^ T responses in the respiratory tract. There appears to be a higher proportion of T cells expressing the cytotoxic protease GzB within the respiratory tract as compared to cytokine-expressing cells. Furthermore, we observed differences in the anatomic localization of these functional T cells (upper versus lower respiratory tract), suggesting that microenvironments within the respiratory tract may influence T cell responses during SARS-CoV-2 infection.

### CD4^+^ and CD8^+^ T cells are dispensable for protection against SARS-CoV-2 in the lungs but required for viral control within the upper respiratory tract

**Fig. 4.**
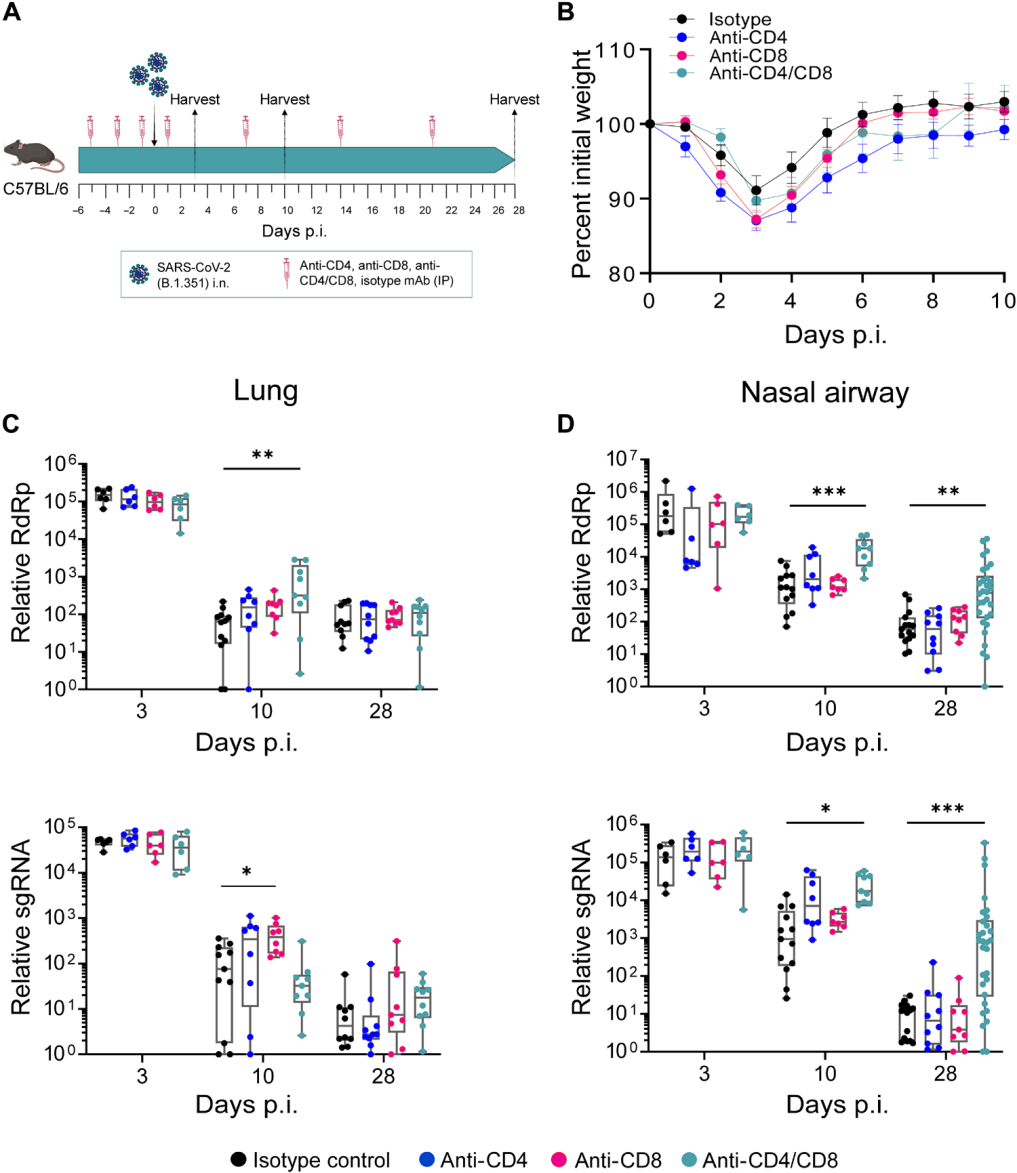
CD4^+^ and CD8^+^ T cells are dispensable for protection against SARS-CoV-2 in the lungs but required for viral control within the upper respiratory tract. (**A**) Study design: C57BL/6 mice were depleted of either CD4^+^ or CD8^+^ or both T cells using 200 μg of anti-mouse CD4 or anti-mouse CD8α or both respectively via intraperitoneal (IP) route at day −5, −3, −1, 1, 7, 14, and 21 p.i. (**B**) Percent initial weight in SARS-CoV-2 (B.1.351) infected mice through 10 days p.i. Viral RNA levels as measured by relative RdRp levels (top) and subgenomic RNA (sgRNA) levels (bottom) at indicated time points in (**C**) lungs (**D**) nasal airways. Graphs show mean ± SEM. Kruskal-Wallis statistical test was performed comparing all depletion groups with the isotype control. *P < 0.05, **P < 0.01, and ***P < 0.001; no symbol, not significant. Data are an aggregate of two independent experiments with group sizes between 6 and 30 mice. i.n., intranasal administration.

To evaluate the contribution of T cells during SARS-CoV-2 infection, we used antibody-based depletion to either individually or tandem deplete CD4^+^ or CD8^+^ T cells (Fig. 4A). Anti-CD4 and/or anti-CD8 antibodies were administered through the intraperitoneal route on days −5, −3, −1, +1, +7, +14, and +21 following SARS-CoV-2 infection. We assessed the efficiency of T cell depletions on days 0 and 28 p.i. and found near complete ablation of CD4^+^ and/or CD8^+^ T cells in whole blood, lungs, and nasal compartment (fig. S4, A and B). Following SARS-CoV-2 infection of CD4^+^ and/or CD8^+^ T cell–depleted mice, we observed similar peak weight loss on day 3 p.i. and recovery through day 10 pi. Further, we observed no mortality in any of the isotype or T cell–depleted SARS-CoV-2–infected mice (Fig. 4B).

We next evaluated virus replication by qRT-PCR (nasal compartment and lungs) and plaque assay (lungs). As a baseline for the depletions at later time points, we assessed viral replication on day 3 p.i. and observed no difference in viral RNA in the nasal compartment or lungs in the isotype or T cell–depleted mice (Fig. 4, C and D). By day 10 pi, we observed a modest, but not statistically significant, increase in viral RNA in the lungs of the individual or tandem CD4^+^/CD8^+^ T cell–depleted mice as compared to the isotype control infected mice. In contrast, viral RNA was higher in the nasal compartment of the CD4^+^/CD8^+^ T cell tandem–depleted mice as compared to the isotype control. In the lungs, by day 28 pi, the T cell–depleted groups showed similar viral RNA as compared to the isotype control infected mice. However, in the nasal compartment, only the CD4^+^/CD8^+^ T cell tandem–depleted mice showed significantly higher viral RNA (30.3-fold) as compared to the isotype control infected mice (Fig. 4, C and D).

To eliminate the possibility that the observed effect on SARS-CoV-2 replication following anti-CD8α antibody depletion was not in any part due to natural killer (NK) cells, we conducted a comparative analysis of the impact of CD8^+^ T cell depletion using either CD8β or CD8α antibodies. Viral RNA levels were assessed in the lungs and nasal airways using qRT-PCR at day 28 p.i. and were compared with the respective individual isotype controls (fig. S5). No difference in viral RdRp levels was detected between the CD8^+^ T cell–depleted mice in either the CD8β or CD8α depletion group, indicating that the persistence of viral RNA at day 28 p.i. in CD4^+^ and CD8^+^ T cell tandem–depleted mice was exclusively attributable to CD8^+^ T cells and not NK cells. These data demonstrate that T cells are required for efficient viral control/clearance within the nasal compartment and, to a lesser extent, within the lungs during SARS-CoV-2 infection. Furthermore, both CD4^+^ and CD8^+^ T cells are required for preventing viral persistence within the nasal compartment and that CD4^+^ and CD8^+^ T cells can compensate for each other in mediating viral control within the nasal compartment.

### SARS-CoV-2 antibody responses are CD4^+^ T cell–dependent but not required for viral control in the respiratory tract

**Fig. 5.**
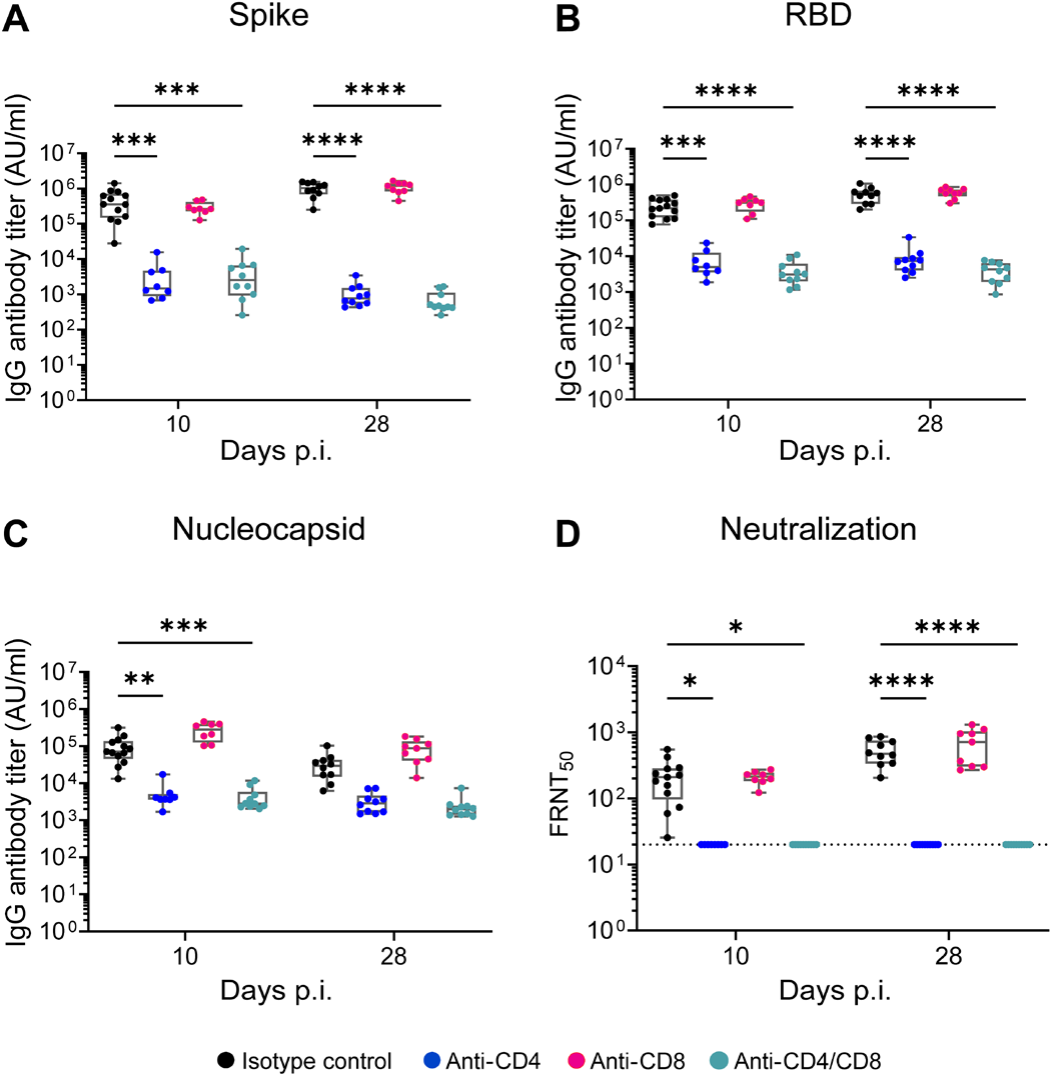
SARS-CoV-2 antibody responses are CD4^+^ T cell–dependent but not required for viral control in the respiratory tract. C57BL/6 mice were depleted of either CD4^+^ or CD8^+^ or both T cells, and at indicated days p.i., binding and neutralizing antibody response against SARS-CoV-2 B.1.351 spike, RBD, and nucleocapsid were measured by electrochemiluminescent multiplex immunoassay and reported as arbitrary units per ml (AU/ml) against SARS-CoV-2. IgG antibody responses were measured against (**A**) Spike, (**B**) RBD, and (**C**) Nucleocapsid. (**D**) The 50% inhibitory titer (FRNT_50_) on the FRNT was measured at day 10 p.i. and day 28 p.i. The dotted line in the FRNT assay represents the maximum concentrations of the serum tested (1/20). A two-way ANOVA statistical test was performed. *P < 0.05, **P < 0.01, ***P < 0.001, and ****P < 0.0001; no symbol, not significant.

Infection and vaccine-mediated antibody responses are essential protection against SARS-CoV-2 infection and have been identified as a correlate of protection (*40*, *41*). To understand how T cells contribute to the antibody response and how these antibodies may control acute SARS-CoV-2 infection, we evaluated binding and neutralizing antibodies. On days 10 and 28 pi, we measured IgG binding antibodies to Spike, RBD, and Nucleocapsid (Fig. 5, A to C). In mice in which CD4^+^ T cells were depleted (individual CD4^+^ or CD4^+^/CD8^+^ tandem depleted), we observed a significant reduction in anti-Spike, anti-RBD, and anti-Nucleocapsid IgG binding antibodies as compared to isotype control. In contrast, we observed no reduction in anti-Spike and anti-RBD IgG binding antibodies in the CD8^+^ T cell–depleted mice. Furthermore, we performed a live virus neutralization assay against B.1.351 and found that CD4^+^ T cell–depleted mice (individual CD4^+^ or CD4^+^/CD8^+^ tandem depleted) showed no neutralizing antibodies above the limit of detection (FRNT_50_ = 20) (Fig. 5D). These findings demonstrate that the antibody response in mice during SARS-CoV-2 infection is CD4^+^ T cell dependent. Further, these data suggest that the antibody response likely plays little to no role in preventing viral persistence in the nasal airways.

### SARS-CoV-2 persists within the nasal epithelium in the absence of CD4^+^ and CD8^+^ T cells

**Fig. 6.**
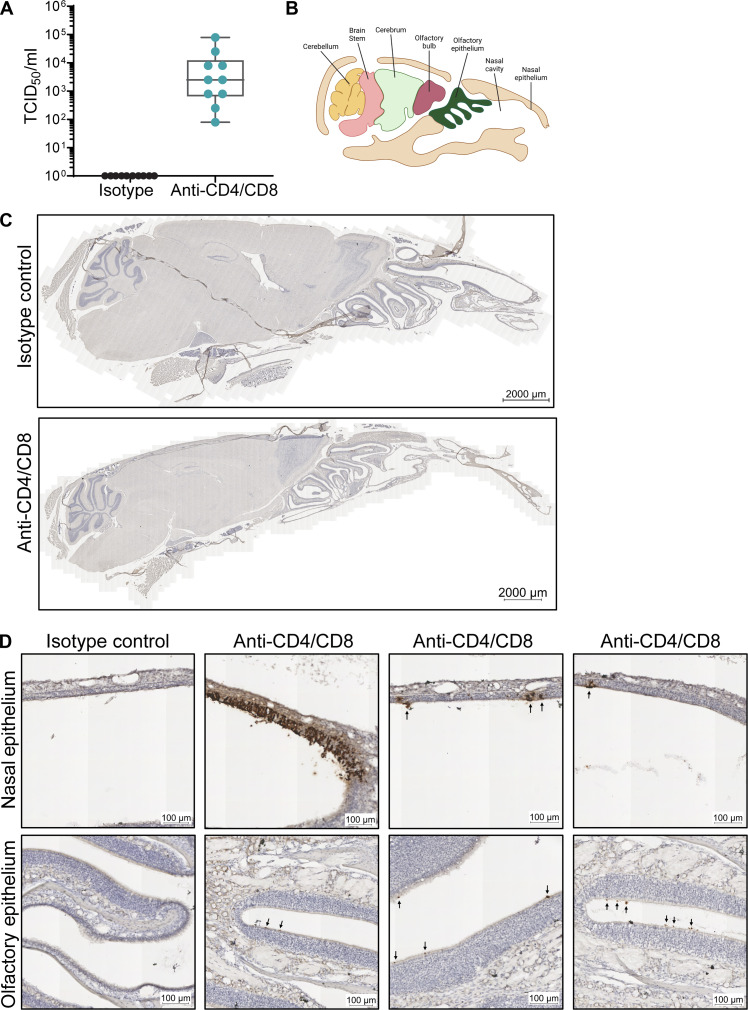
SARS-CoV-2 persists predominantly within the nasal epithelium in the absence of CD4^+^ and CD8^+^ T cells. C57BL/6 mice were depleted of both CD4^+^ or CD8^+^ and assessed for localization of viral antigen in mouse heads at 28 days p.i. (**A**) Tissue culture infectious dose (TCID_50_) per milliliter of nasal turbinate suspension assessed in mice where both CD4^+^ and CD8^+^ T cells were depleted. (**B**) Representation of the sagittal section of a mouse skull showing various parts of the nasal cavity and brain (created with BioRender.com). (**C**) Representative images of ISH for RNA Spike antigen performed on heads of mice where both CD4^+^ and CD8^+^ T cells were depleted and compared to isotype control mice. (**D**) Representative images of nasal epithelium (top) and olfactory epithelium (bottom) of ISH for RNA Spike antigen. Arrows represent anti-spike RNA (dark brown). Representative images from 3 of 10 T cell–depleted mice.

To understand whether the persistent viral RNA in the nasal airways corresponds to infectious virus or not, we performed a median tissue culture infectious dose (TCID_50_) infectious virus assay from nasal compartment suspensions of isotype and CD4^+^/CD8^+^ tandem–depleted mice on day 28 p.i. (Fig. 6A). We found that 10 of 10 mice showed infectious virus from the tandem-depleted infected mice, but not the isotype control infected mice, with an average viral titer of 1.26 × 10^4^ TCID_50_/ml. To determine where the virus is replicating within the nasal compartment, we performed ISH using RNAscope on the entire head of a mouse, which includes the nasal compartment, olfactory bulbs, and brain, with a spike-specific RNA probe (Fig. 6, A and C). We observed no viral RNA staining within brain or olfactory bulbs in either the isotype control (*n* = 5) or tandem-depleted mice (*n* = 10), suggesting that in the presence or absence of T cells, SARS-CoV-2 does not infect the brain. CD4^+^/CD8^+^ T cell tandem–depleted mice, but not isotype control mice, consistently showed viral RNA staining within the nasal compartment. We observed viral RNA staining within the epithelial layer and infection with little replication within the basement membrane. In addition, infection was more commonly seen in the nasal epithelium than the olfactory epithelium found in the ethmoturbinates (Fig. 6D). These findings demonstrate that both CD4^+^ and CD8^+^ T cells are required to prevent viral persistence within the nasal epithelium.

### Viral persistence leads to increased viral diversity

In humans, persistent SARS-CoV-2 infection of immunocompromised individuals can lead to increased viral genetic diversity (*42*–*46*). To understand the impact of viral persistence on SARS-CoV-2 evolution, we isolated virus on day 28 p.i. from the nasal turbinates of CD4^+^/CD8^+^ tandem–depleted mice and propagated it once using Vero-TMPRSS2 cells, which we have shown previously to minimize genetic diversity during virus isolation and propagation (*47*). We refer to these samples as “isolate-28d.” Concurrently, the B.1.351 stock virus was also cultured in Vero-TMPRSS2 cells (denoted: “inoculum-Vero”) to identify changes that may occur during a single viral passage. The inoculum-Vero and stock virus used to infect the mice (“inoculum-stock”) serve as the baseline for virus diversity. These sequences were compared with the stock virus used to infect the mice, referred to as inoculum-stock in the text. To account for sequence mutations resulting from mouse adaptations, mice were also infected with inoculum stock virus and samples obtained from nasal turbinates on day 3 p.i. (referred to in this text as “inoculum-3d”) where we observe peak viral titers (Fig. 1B). These mouse isolates and inoculum controls were sequenced to characterize emergent intrahost SARS-CoV-2 variants.

**Fig. 7.**
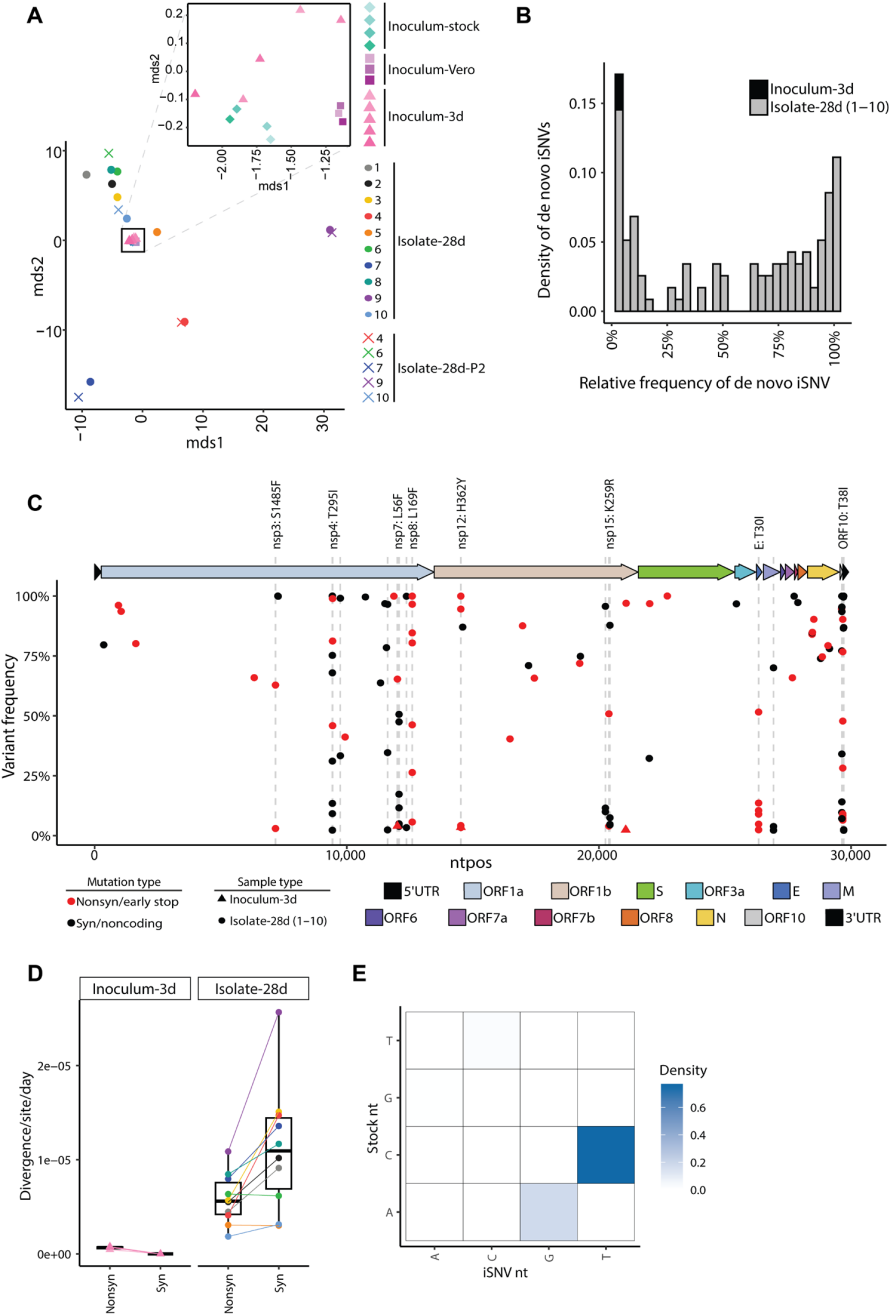
Intrahost SARS-CoV-2 variants emerge during infection in tandem CD4^+^/CD8^+^ T cell–depleted mice. (**A**) MDS plot of the pairwise genetic distance (L1-norm) calculations across all samples in the dataset. The inset zooms in on the black box to show the positions of the inoculum samples. Point color and shape represent the sample and collection type. For the inoculum-stock, the color gradient represents different aliquots of the stock. For inoculum-Vero and inoculum-3d, the color gradients represent different infections. (**B**) The frequency distribution of de novo variants identified in the inoculum-3d (black) and isolate-28d (1 to 10, gray) samples. (**C**) The location, frequency, and characteristics of de novo variants in the inoculum-3d (triangle) and isolate-28d (1 to 10, circle) samples. The color of each point represents the type of mutation including mutations that are synonymous/in noncoding regions (black) or nonsynonymous/stop codon mutations (red). Point shape indicates the type of sample collection. Dashed lines highlight the genomic positions where a de novo mutation was found in more than one mouse isolate-28d sample. Labels are added for the nonsynonymous substitutions only. The colors on the genome map represent the different gene regions. 5′UTR, 5′ untranslated region; ORF, open reading frame; S, spike; E, envelope; M, membrane; N, nucleocapsid; nsp, nonstructural protein. (**D**) The nonsynonymous and synonymous divergence rates normalized by the expected number of sites in the coding sequence for the inoculum-3d samples and T cell–depleted mouse isolate-28d samples. Point color and shape represent the sample and collection type as outlined in Fig. 7A. (**E**) The density of transitions and transversions for all de novo mutations (2 to 100%). nt, nucleotide.

To quantify the variation in the virus populations between the mouse isolates-28d and inoculum controls, we calculated the pairwise genetic distance using the L1-norm. All the three controls (the inoculum-Vero, inoculum-3d, and inoculum-stock) were similar in diversity to each other (Fig. 7A) and differed only slightly likely due to variations in their low-frequency minor variant populations (Fig. 7A, inset, and fig. S6). Compared to the inoculum-3d controls, all virus populations in the mouse isolates significantly diverged from the inoculum-stock (*P* value = 6.49 × 10^−16^).

To identify mutations that emerged de novo in the tandem T cell depleted mice by day 28 pi, we filtered for intrahost single-nucleotide variants (iSNVs) that were not present in the standing diversity of the inoculum-stock and inoculum-Vero samples (0.5 to 100%). The location, number, and frequency of de novo iSNVs varied by mouse isolate (Fig. 7, B and C), with isolate 10 having zero iSNVs that reached ≥50% while isolate 9 had 15 (6 of which were amino acid substitutions) (fig. S7). The ORF10, nsp7, and envelope had the highest mutational densities with averages of 14.5 (±5.8), 3.2 (±2.5), and 2.6 (±2.3) de novo iSNVs/kb, respectively. Of the 57 unique de novo mutations, 19 emerged in more than one mouse, 8 of which generated amino acid substitutions in the ORF1a (nsp3, nsp4, nsp7, and nsp8), ORF1b (nsp12 and nsp15), E, and ORF10 gene regions. All 19 mutations were present in a small subset (<1%) of globally circulating SARS-CoV-2 sequences (CoV-Spectrum 01 June 2020 to 12 May 2023) (*48*). In addition, the nsp4 T295I mutation has previously been reported in mouse-adapted strains of SARS-CoV-2 (*17*). The remaining nonsynonymous mutations have not been characterized, although others have identified mouse-specific adaptations that emerged in nsp6, nsp7, and nsp8 (*16*, *49*). iSNVs shared in more than one mouse isolate were more likely to be present as minor variants (<50%) compared to iSNVs found in only a single mouse isolate (*P* value = 0.0003, Fisher’s exact), indicating that the shared iSNVs may emerge at different points during infection.

In the mice where both CD4^+^ and CD8^+^ T cells were depleted, the synonymous divergence rates (mean = 1.12 × 10^−05^ per site per day) were higher than the nonsynonymous (mean = 5.84 × 10^−06^ per site per day) (Fig. 7D, *P* value = 0.043, Mann-Whitney *U*), indicating that purifying selection is occurring in the CD4^+^/CD8^+^ tandem–depleted mice. Further, the rates observed in our mouse isolates fall into similar ranges observed in immunocompromised individuals with prolonged infections (≥21 days) (*50*). More than 76% of the iSNVs were C-to-T transitions (Fig. 7E), a signature of SARS-CoV-2 evolution observed in human intrahost studies and globally circulating strains (*51*).

### Genetic diversity within SARS-CoV-2 leads to reduced virus replication in the lungs

**Fig. 8.**
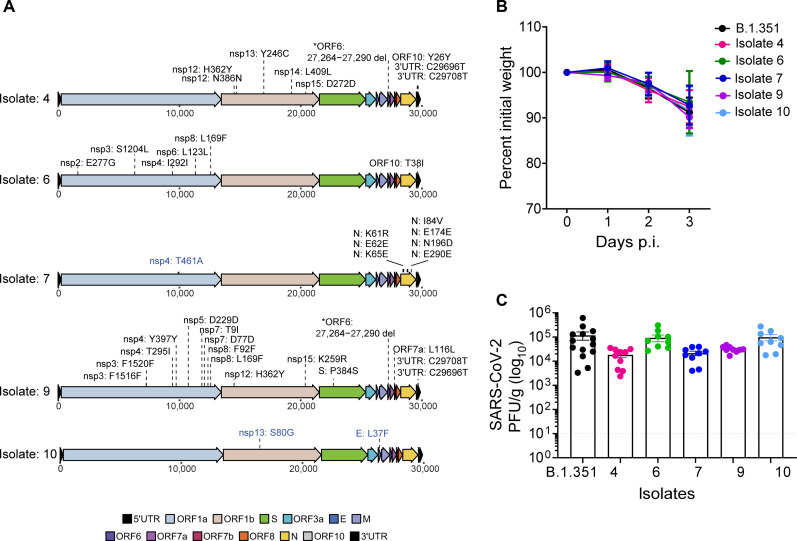
De novo mutations lead to differences in virus replication. (**A**) Mutation maps of de novo SARS-CoV-2 consensus mutations (≥50%) in the mouse isolates 4, 6, 7, 9, and 10 and compared to the isolate-Vero samples. Blue labels represent mutations that reached ≥50% in the isolate-Vero sample but were present at <50% in the original mouse isolate. Labels represent amino acid information for mutations in coding regions and nucleotide information for deletions or mutations in the noncoding regions of the genome. The colors on the genome map represent the different gene regions. (**B**) C57BL/6 mice were infected with mice isolates-P2 as indicated, and weight loss was measured over 3 days. The graph represents the percent initial weight compared to the initial weight on the day of infection. (**C**) Infectious virus from lungs of mice infected with indicated isolates at day 3 p.i. was quantified by plaque assay in VeroE6-ACE2-TMPRSS2 overexpressing cells and expressed as PFU per gram of lung tissue.

We next selected five mice isolates based on their distance from the inoculum-stock and de novo iSNV populations (Fig. 7A and fig. S8A). Isolates 4, 7, and 9 diverged from the inoculum-stock, the most with 13, 20, and 21 de novo iSNVs, respectively (fig. S6). Isolates 4 and 9 had a consensus level deletion at nucleotide positions 27,264 to 27,290 (amino acids 22 to 30) in ORF6 (fig. S8A). This deletion was also found at <1% in the inoculum-stock (fig. S8B). Isolate 6 had the highest number of mutations in the ORF6 region. Isolate 7 had de novo mutations that emerge in only the nucleocapsid gene (Fig. 8A). Isolate 10 had zero iSNVs that reached ≥50% and was diverged the least from the inoculum-stock. We propagated these five isolates on VeroE6-TMPRSS2 cells (“isolate-28d-P2”) to generate working in vivo stocks. After sequencing the isolate-28d-P2 samples, we confirmed that no new mutations emerged upon viral propagation except for one mutation in isolate 7 (nsp4: T461A), and two mutations in isolate 10 (nsp13: S80G and E: L37F) increased in frequency after propagation.

We next infected C57BL/6 mice with the five mice isolates-28d-P2 at 10^6^ PFU/ml and measured weight loss throughout the infection and infectious viral titers in the lungs at day 3 pi. We observed similar weight loss dynamics across the five isolates compared to the mice infected with the B.1.351 stock virus (Fig. 8B). Isolate 10, which was the most similar to the stock B.1.351 virus, had similar titers to B.1.351-infected mice (Figs. 7A and 8C). The most divergent isolates, 4, 7, and 9, showed a 6.44-, 5.44-, and 3.71-fold reduction, respectively, in virus replication in the lung as compared to B.1.351-infected mice (Figs. 7A and 8C). Collectively, these findings demonstrate that viral persistence leads to increased SARS-CoV-2 intrahost genetic diversity, and these changes can lead to differences in virus replication at early times following virus infection.

## DISCUSSION

The magnitude and quality of the T cell response is essential for driving protection against SARS-CoV-2 infection as well as promoting vaccine efficacy (*52*). Several groups have investigated T cell responses against SARS-CoV-2 infection in the lungs (*52*–*56*); however, the contribution of these cells in promoting viral control and clearance within the upper respiratory tract is not well understood. In our study, we demonstrate that T cells are necessary for controlling virus replication in the nasal airways, but not the lungs, during SARS-CoV-2 infection. Using antibodies to deplete T cells before infection, we found that CD4^+^ and CD8^+^ T cells play distinct roles in the upper and lower respiratory tract. Tandem depletion of both CD4^+^ and CD8^+^ T cells, but not individual, results in persistent virus replication in the nasal compartment through 28 days p.i. The persistent virus was culturable as we were able to recover infectious virus from the nasal compartment of all tandem-depleted mice nearly a month after infection. Further, we used ISH to determine that the virus was replicating within the nasal epithelium, and not the olfactory bulbs or brain, during persistent infection. Through deep sequencing, we found that the virus isolates from persistently infected mice show mutations across the viral genome, with several mice showing deletions within the ORF6 gene. Combined, these findings highlight the importance of T cells in controlling virus replication within the respiratory tract during SARS-CoV-2 infection.

Our studies show that T cells are critical for viral clearance within the upper respiratory tract, especially within the nasal compartment but not the lungs. In support, previous studies with the related SARS-CoV-1 virus found that B and T cells were dispensable for controlling virus replication within the lungs, highlighting the importance of innate immunity within the lungs for viral control against coronaviruses (*57*). Our data also support an important role for the innate response in controlling virus replication within the respiratory tract. Studies have found that type I and III IFNs can control SARS-CoV-2 replication in the upper and lower respiratory tracts (*58*, *59*). Further, plasmacytoid dendritic cells (pDCs) and alveolar macrophages have been implicated in modulating inflammation and viral control (*60*–*63*). We recently determined the importance of the CCR2-monocyte signaling axis in promoting virus control and dissemination within the lungs and mediating protection (*22*). In addition to innate immune cells, it is also plausible that innate-like lymphocytes such as innate-like B and T cells such as B-1 cells, NKT cells, γδ T cells, and mucosal-associated invariant T cells may also play a role in clearing the SARS-CoV-2 virus from the lungs for which the field is still beginning to evolve (*64*–*68*). Together, these studies show that a combination of the innate, adaptive, and innate-like adaptive immune response is important for controlling virus replication, mitigating viral dissemination, limiting inflammation, and protecting against lethal disease outcome.

One interesting observation was that neutralizing antibodies do not appear to play an important role in controlling SARS-CoV-2 infection. First, we show that depletion of CD4^+^ T cells dramatically reduces SARS-CoV-2–specific spike and nucleocapsid binding antibodies. This is consistent with studies in humans which have shown that antibody responses during SARS-CoV-2 infection and vaccination are T cell dependent (*24*, *69*, *70*). Second, we show that the lack of CD8^+^ T cells does not alter SARS-CoV-2 spike binding or neutralizing antibody responses. Last, we show similar control of virus replication within the upper and lower respiratory tracts on day 10 and 28 p.i. in the absence of either CD4^+^ or CD8^+^ T cells, suggesting that antibody response is not a major driver of viral control within the respiratory tract during infection. It is possible that nonneutralizing and neutralizing antibodies play a subtle role in the kinetics of viral clearance within the respiratory tract, and future studies should specifically look at the timing of the antibody response and viral control within the respiratory tract.

Under normal conditions, following respiratory virus infection, effector CD4^+^ and CD8^+^ T cells are primed in the lung-draining lymph nodes and then traffic back to the infected lung to control virus replication through their effector mechanisms (*52*). There are many factors that can influence T cell programming, including but not limited to the local cytokine environment, engagement with antigen-presenting cells (APCs), interaction with costimulatory molecules, and antigen load. Our findings suggest that T cell priming may be different between the upper and lower respiratory tract. While GzB-secreting T cells were similar between the nasal airways and lungs, we did observe differences in cytokine-producing T cells. This suggests that anatomic compartmentalization could influence T cell responses within the respiratory tract during SARS-CoV-2 infection. Analogously, we have found during West Nile that virus infection, an encephalitic RNA virus that targets the brain, shows differences in T cell activation and effector functions between the brain and spleen (*71*). In the context of LCMV infection, we have previously shown that anatomic location is a driver of memory precursor CD8^+^ T cells into long-term memory cells within the white pulp region of the spleen (*72*). Future studies should focus on identifying APC–T cell interactions within the lung-draining lymph node and within the lungs as well as how the inflammatory milieu may influence T cell responses within the upper and lower respiratory tract.

In individuals with weakened immune systems, the clearance of viruses may be delayed, resulting in prolonged shedding of the virus and accumulation of mutations in the virus genome (*42*, *73*). One leading hypothesis is that chronic infections in immunocompromised individuals are an important source of SARS-CoV-2 genetic diversity, driving the emergence of highly divergent SARS-CoV-2 variants of concern (*42*, *43*). However, data are limited for studying how virus persistence, antiviral therapeutics, and host response during chronic infections affect the intrahost evolution of SARS-CoV-2. In our study, we detected viral RNA persisting in the nasal airways and successfully isolated and sequenced infectious virions from the nasal turbinates 28 days p.i. Sequence analysis of the virus populations collected from persistent infections revealed de novo mutations that accumulated at high frequencies across the viral genome, including mutations in ORF10 and ORF6. ORF10 had the highest density of mutations and has been shown to degrade antiviral innate immunity by mitophagic degradation of mitochondrial antiviral signaling (MAVS) (*74*). We observed a 27-nt-long deletion in ORF6 that was present at <1% in the stock inoculum but reached >50% in mouse isolates 4 and 9. The significance of the ORF6 region in antagonizing IFN signaling during SARS-CoV-1 infections is well-established. However, conflicting findings exist regarding the role of ORF6 in SARS-CoV-2 infection, with studies showing both direct interference with IFN signaling through binding to nucleoprotein Nup98 and contradictory outcomes in animal models, including mice and hamsters (*75*–*79*). There was limited diversity found in the Spike protein. Among the three observed Spike mutations, only one was present in the receptor binding domain (P384S, isolate 9), and of the 19 mutations shared, only one (nsp4 T295I) has been identified as a mouse adaptation. Our findings, which include elevated rates of synonymous mutations and a higher occurrence of C-to-T transitions, align with observations made in prolonged SARS-CoV-2 infections of immunocompromised humans (*44*, *51*, *80*). These studies collectively highlight how mutations within the viral genome can result in strategies to evade the immune system, underscoring the importance of thorough investigation into these mutations. This viral persistence model, which leads to viral sequence changes, offers a valuable tool for exploring intrahost evolutionary dynamics of SARS-CoV-2.

In summary, our studies highlight the importance of T cells in mediating viral control within the respiratory tract. These findings directly affect the future design of mucosal vaccines and demonstrate the importance of promoting T cell immunity within the upper and lower respiratory tract. Further, these studies now provide a model to study innate immunity and virus-host interactions in the context of viral persistence within the upper respiratory tract especially the nasal compartment.

## MATERIALS AND METHODS

### Study design

The study design involved multiple experimental approaches to investigate the immune response and genetic changes associated with Beta variant infection in mice. C57BL/6J mice were infected with Beta variant intranasally and monitored for clinical pathology and mortality p.i. Flow cytometry analysis of immune cell populations was performed to evaluate tissue-specific immune responses, and ex vivo T cell assays were conducted to measure antigen-specific T cell responses. Antibody binding assays, focus reduction neutralization assays, qRT-PCR, and TCID_50_ assays were used to assess antibody responses and viral titers. T cell depletion experiments were conducted using anti-CD4 and anti-CD8α antibodies administered intraperitoneally at specific time points p.i. ISH of brain tissues was performed on mouse heads to determine the location of persistent virus observed in CD4/DC8 tandem–depleted mice. Genetic analysis was performed using Illumina sequencing and single-nucleotide variant analysis. Genetic distance and divergence calculations were used to evaluate viral evolution.

### Viruses and cells

VeroE6-TMPRSS2 cells were generated as previously described (*81*) and cultured in complete Dulbecco’s modified Eagle’s medium (DMEM) consisting of 1× DMEM (VWR, #45000-304), 10% fetal bovine serum (FBS), 2 mM l-glutamine, and 1× antibiotic in the presence of puromycin (10 mg/ml; Gibco). The SARS-CoV-2 B.1.351 variant, obtained from A. Pekosz at John Hopkins University in Baltimore, MD, was plaque purified, propagated in VeroE6-TMPRSS2 cells to create a working stock, and sequence-confirmed. Viral titers were determined through plaque assays conducted on VeroE6-TMPRSS2-hACE2 cells (provided by B. Graham, Vaccine Research Center, National Institutes of Health, Bethesda, MD) as described here (*22*).

### Mouse experiments

C57BL/6J mice were purchased from the Jackson Laboratory or bred in-house at the Emory National Primate Research Center rodent facility at Emory University. All mice used in these experiments were females between 8 and 12 weeks of age. Mice were anesthetized using isoflurane and infected with SARS-CoV-2 B.1.351 at 10^6^ PFU intranasally in a final volume of 50 μl in saline in accordance with the institutional standard operating procedure for working in animal biosafety level 3 facility. After infection, mice were monitored daily for any clinical pathology and mortality. For experiments involving T cell depletion, mice were administered 200 μg per mouse of either anti-CD4 (clone GK1.5, BioXCell) or anti-CD8α (clone 2.43, BioXCell), or a combination of both anti-CD4 and anti-CD8α antibodies, or an isotype monoclonal antibody (mAb) IgG1 (clone HRPN, BioXCell). The administration was done via the intraperitoneal route on days −5, −3, −1, +1, +7, +14, and +21 following SARS-CoV-2 infection. Unless otherwise specified, all CD8^+^ T cell depletions were carried out using the anti-CD8α antibody. The impact of CD8^+^ T cell depletions on SARS-CoV-2 infection was also evaluated using anti-CD8β (clone 53-5.8, BioXCell) and isotype mAb IgG2b (clone LTF-2, BioXCell). All experiments and the mouse handling and care procedures followed the guidelines of the Emory University Institutional Animal Care and Use Committee (PROTO201700309).

### Antibody binding assays

Serum collected from infected mice was tested to assess the binding of IgG antibodies against B.1.351 spike, RBD, and nucleocapsid using the V-PLEX SARS-CoV-2 Panel 7 (Mouse IgG) Kit [Meso Scale Discovery (MSD), #K15484U-2] per the manufacturer’s protocol (*81*). Briefly, plates coated with the specific SARS-CoV-2 antigens were blocked using MSD blocker at room temperature, with shaking at 700 rpm for 30 min. Samples were diluted 1:1000 and incubated with the plates for 2 hours at room temperature. Following this, SULFO-TAG conjugated goat anti-mouse IgG antibody was added. The plates were washed with 1× MSD Wash Buffer, and then MSD Gold Read Buffer B was added to each well. Between each step, the plates were washed three times with phosphate-buffered saline (PBS) containing 0.05% Tween. An MSD plate reader was used to read the plates, and the results were analyzed using Discovery Workbench software, version 4.0, and reported as arbitrary units per ml (AU/ml) against SARS-CoV-2.

### Focus reduction neutralization assay

FRNT assays were conducted following the methods outlined in the protocol described previously (*82*). In summary, the samples were diluted in threefold increments, creating eight serial dilutions using DMEM in duplicates. The initial dilution was set at 1:10, and the total volume reached 60 μl. The serially diluted samples were then incubated with virus at 37°C for 1 hour in a round-bottomed 96-well culture plate. After the incubation, the antibody-virus mixture was added to Vero-TMPRSS2 cells and incubated again at 37°C for 1 hour. Following this step, the antibody-virus mixture was removed, and a 0.85% methylcellulose overlay (Sigma-Aldrich) was added to each well. The plates were further incubated at 37°C for 18 hours. Once the incubation was complete, the methylcellulose overlay was removed, and cells were washed with PBS and fixed using 2% paraformaldehyde in PBS. Plates were washed with PBS and permeabilized using a buffer containing 0.1% bovine serum albumin and Saponin in PBS for 20 min. Following this, cells were incubated overnight at 4°C with the anti-SARS-CoV spike primary antibody directly conjugated to Alexa Fluor 647 (CR3022-AF647). Subsequently, the cells underwent two washes with 1× PBS before imaging on an ELISPOT reader (Cellular Technology Limited analyzer).

### TCID_50_ assay

VeroE6-TMPRSS2 cells were seeded at a density of 25,000 cells per well in complete DMEM media in quadruplicates for each sample. Once confluent, the medium was removed, and 180 μl of DMEM containing 2% FBS was added. Serial dilutions of samples, along with positive controls (virus stock with a known infectious titer) and negative controls (medium only), were included. The plates were further incubated at 37°C with 5.0% CO_2_ for 2 to 5 days. Cells were fixed and stained with a crystal violet solution containing 2% paraformaldehyde. Visual inspections were carried out on cell monolayers to detect any cytopathic effect, and TCID_50_ was determined using the Read-Muench formula (*83*).

### ISH of brain tissues

Mouse heads collected on day 28 p.i. were subjected to decalcification in EDTA for approximately 2 weeks, followed by rehydration in PBS for 2 days. Subsequently, the samples were immersed in a 30% sucrose solution prepared in PBS for 3 to 4 days to achieve sucrose equilibration. Once equilibrated, the heads were rapidly frozen in 100% optimal cutting temperature (OCT) compound and stored at −80°C. Tissues were sliced using a cryostat and stored at −80°C. RNA ISH was performed following the RNAscope Brown Kit protocol, with a modification for OCT-frozen tissues where a tissue is postfixed onto the slide. The tissue was stained for Spike RNA using a custom RNAscope Probe V-nCoV2019-S (Advanced Cell Diagnostics, #848561). Images were obtained using a Zeiss AxioImager Z2 system with Zeiss software at 20× on the Slide Scanner, an automated imaging system.

### Processing of mouse tissues for flow cytometry analysis

On the specified day following infection, mice were anesthetized using isoflurane and given a retro-orbital injection of CD45:PE (BD Biosciences, clone 30-F11) diluted 1:20 in saline in a final volume of 100 μl per mouse. After a 5-min recovery period, the mice were euthanized using isoflurane overdose. Lung, URT tissues, and spleens were harvested from each mouse and placed in 1% FBS–Hanks’ balanced salt solution (HBSS). The spleens underwent mechanical homogenization on a 70 μM cell strainer, and the resulting cell suspension was collected in 10% FBS-RPMI. Splenocytes were processed by centrifugation (1250 rpm, 5 min, 4°C), followed by lysis in ammonium–chloride–potassium (ACK) lysis buffer for 5 min on ice. After washing with 10% FBS-RPMI, the splenocytes were kept chilled until they were ready for further applications. The lung and URT tissues were mechanically disrupted in six-well plates and then subjected to a 30-min digestion at 37°C in a solution of deoxyribonuclease I and collagenase in HBSS. The digestion was halted with 10% FBS-RPMI, and the lung cells were filtered through a 70 μM filter to obtain a single-cell suspension. The cells obtained from lung samples were then layered onto a Percoll-PBS gradient and centrifuged, and the top layer of cell debris was removed. The resulting cell pellet was lysed with ACK lysing buffer for 5 min on ice, followed by washing and resuspension in 10% FBS-RPMI, keeping the cells chilled until they were ready for the staining process. The URT samples were directly lysed with ACK lysing buffer, washed, and resuspended in 10% FBS-RPMI until further processing for flow cytometry analysis.

### Flow cytometry analysis

Single-cell suspensions were centrifuged and were then resuspended in a blocking solution containing anti-CD16/32 (Tonbo, clone 2.4G2) for 20 min at 4°C. After centrifugation, the cell suspensions were stained using surface stain antibodies including Live/Dead Ghost Dye stain (Tonbo) for 20 min at 4°C. Following this, the stained cells were washed and fixed in 2% paraformaldehyde-PBS for 30 min at room temperature. Last, cells were washed and resuspended in 300 μl of fluorescence-activated cell sorting (FACS) buffer (1% FBS in 1× PBS). Precision count beads (BioLegend) were added to the samples to facilitate cell counting. These samples were then processed using a BD FACS Symphony A5 instrument. The anti-mouse surface staining antibodies used in this study were as follows: CD45:PE (BioLegend, clone: 30-F11), CD45.2:BV650 (BioLegend, clone: 104), CD44:FITC (BioLegend, clone: NIM-R8), CD3e:PerCP Cy5.5 (Tonbo, clone: 145-2C11), interleukin-7R (IL-7R):PE/cyanine 5 (BioLegend, clone: A7R34), KLRG1:APC-Cy7 (BioLegend, clone: 2F1), CD8b:BV421 (BioLegend, clone: YTS156.7.7), Live/Dead Ghost Dye UV 450 (Tonbo), CD69:BV785 (BioLegend, clone: H1.2F3), CD103:AF700 (BioLegend, clone: 2E7), and CD4:PE-Cy7 (BioLegend, clone: GK1.5).

### Ex vivo T cell assays

For T cell peptide restimulation, approximately 1 × 10^6^ cells harvested from tissues were placed per well in a 96-well round-bottom plate and stimulated for 6 hours at 5% CO_2_, 37°C, with the addition of brefeldin A (BFA; 10 μg/ml) in complete RPMI media. For positive control, splenocytes were stimulated with BFA and phorbol 12-myristate 13-acetate/ionomycin. For negative control, cells were stimulated with BFA and vehicle dimethyl sulfoxide in the same media. To measure antigen-specific T cell responses, splenocytes, URT, and lung cells were stimulated with SARS-CoV-2 spike peptide pool (1 μg/ml; Biodefense and Emerging Infections Research Resources Repository) in the presence of BFA. After T cell stimulation, cells were washed with FACS buffer and stained for the surface antigens as described in the previous section. Cells were then washed with FACS buffer and incubated with 1× Fix/Perm solution (Tonbo) at room temperature for 1 hour. Following this, cells were washed with 1× Perm buffer (Tonbo) and stained for intracellular antigens for 30 min at 4°C with the following antibodies: GzB:AF647 (BioLegend, clone: GB11), IL-2:BV605 (BioLegend, clone: JES6-5H4), TNFα (BioLegend, clone: MP6-XT22), and IFN-γ:PE Dazzle 594 (BioLegend, clone: XMG1.2). Following intracellular staining, cells were washed twice with the 1× Perm buffer and once with FACS buffer. Cells were resuspended in 300 μl of FACS buffer. Precision count beads (BioLegend) were added to the samples to facilitate cell counting. These samples were then processed using a BD FACS Symphony A5 instrument. For data analysis, splenocytes were gated on lymphocytes, single cells, live cells, parenchymal lymphocytes, and CD3^+^ and then further categorized as either CD4^−^, CD8^+^ for CD8 T cell response analysis or CD4^+^, CD8^−^ for CD4 T cell analysis. Antigen-specific cells were identified on the basis of their production of IFN-γ, TNF-α, or both cytokines in response to SARS-CoV-2 peptide restimulation.

### Quantitative reverse transcription polymerase chain reaction

To prepare tissue samples for evaluating viral RNA levels and mRNA expression, lung and nasal turbinate tissues were collected in an Omni Bead Ruptor tube containing Tri reagent (Zymo). Subsequently, tissues were homogenized using the Omni Bead Ruptor 24 instrument with a program set at 5.15 m/s for 15 s. The samples were then stored at −80°C until further analysis. Samples in Tri reagent were briefly spun, then RNA was extracted using a Direct-zol RNA miniprep kit (Zymo), and cDNA was prepared using the High-Capacity cDNA Reverse Transcription Kit (Thermo Fisher Scientific) as per the manufacturer’s protocol. Viral RNA levels and replication were measured as previously described (*58*). Briefly, qRT-PCR was set up using Integrated DNA Technologies, Inc. (IDT) Prime Time gene expression master mix on a QuantStudio5 qPCR system using the cycling conditions recommended by the manufacturer. To measure viral RNA levels, SARS-CoV-2 RdRp-specific forward primer (GTGARATGGTCATGTGTGGCGG), reverse primer (CARATGTTAAASACACTATTAGCATA), and probe 56-6-carboxyfluorescein [FAM]/CAGGTGGAA/ZEN/CCTCATCAGGAGATGC/3IABkFQ were used. To measure virus replication, levels of SARS-CoV-2 E gene subgenomic RNA was measured using forward primer sgLeadSARSCoV2-F: 5′-CGATCTCTTG-TAGATCTGTTCTC-3′ (IDT) and the E_Sarbeco R2 reverse primer (IDT, #10006890) and P1 FAM probe (IDT, #10006892). Glyceraldehyde phosphate dehydrogenase (GAPDH) was used as a reference gene to normalize viral RNA levels which were represented as fold change over mock samples.

### Illumina library preparation, sequencing, and alignment

SARS-CoV-2 RNA was isolated using the RNAzol RT Column Kit (Molecular Research Center Inc.) as per the manufacturer’s instructions from the B.1.351 stock samples (*n* = 4, inoculum-stock), B.1.351 stock samples passaged once in VeroE6-TMPRSS2 cells (*n* = 3, “inoculum-Vero”), nasal turbinates of C57BL/6 control mice at day 3 p.i. (*n* = 5, inoculum-3d), nasal turbinates of CD4^+^/CD8^+^ tandem–depleted mice on day 28 p.i. and passaged once in VeroE6-TMPRSS2 cells (*n* = 10, “isolate-28d”), and five mouse isolates passaged once more in VeroE6-TMPRSS2 cells (isolate-28d-P2). Approximately 400-bp-long amplicons were generated from the isolated SARS-CoV-2 viral RNA using the ARTIC V4 primers and protocol (https://artic.network/2-protocols.html). Amplicons were cleaned using AMPure beads and input into the Illumina DNA Prep Kit (Illumina, San Diego, CA) according to the manufacturer’s protocol. The concentration and fragment size of the libraries were determined using the Qubit dsDNA high-sensitivity assay (Thermo Fisher Scientific, Waltham, MA) and a high-sensitivity D1000 screentape (Agilent, Santa Clara, CA), respectively. The final libraries were pooled at equal molarity and sequenced on the MiSeq (v3 600 cycles, Illumina, San Diego, CA). Amplification, library preparation, and sequencing were done twice on the same RNA sample.

Reads were trimmed with trimmomatic v0.39 (*84*) and aligned to the Wuhan/Hu-1 SARS-CoV2 genome (NC_045512.2) using bwa mem v0.7.17 (*85*). ARTIC v4 primer sequences were removed using iVar v1.3.1 (*86*) with a minimum quality threshold of 0, including all reads with no primer sequences found. Consensus sequences and variants were called using an in-house variant calling pipeline, timo (v4) (https://github.com/GhedinLab/timo) (*87*). The amplification and library preparation protocol and alignment pipeline are available at https://github.com/GhedinSGS/SARS-CoV-2_analysis. The sequencing data are available at Sequence Read Archive (PRJNA1064978).

### Single-nucleotide variant analysis

Coverage and variant data were pulled from the timo outputs (fig. S5, A to C). Minor variants in the inoculum and inoculum-Vero samples were required to be present in both sequencing replicates with an average frequency of 0.5 to 49% and read depth (dp) of 200×. Nucleotides ≥ 50% at positions with ≥10× dp were considered the consensus nucleotide for the sample. All consensus sequences for the inoculum-stock and inoculum-Vero samples were confirmed to be identical. Passaging the inoculum stock once in the VeroE6-TMPRSS2 filtered out a large proportion of low-frequency (mean = 1%, median = 0.7%) minor variants in the inoculum-stock used for infections (fig. S5, D and E). Therefore, iSNVs that were generated de novo were required to be present at 2 to 49% and at positions with 200× dp for minor variants or 50 to 100% 10× dp for consensus variants and not present in any inoculum stock or inoculum-Vero samples, including as a minor variant at 0.5 to 49%. Tables outlining the de novo iSNVs are located at https://github.com/GhedinSGS/TCD_Mice.

### Genetic distance and divergence calculations

Divergence rates were calculated as previously outlined (*88*). The number of nonsynonymous and synonymous sites was estimated for each sample using the number of nucleotide positions in the coding sequence with at least 200× dp. All mouse isolates had >90% coverage of the coding sequence at 200×. Sites that lacked any minor variant and were identical in their consensus nucleotide across all samples were not used for distance calculations, as the distance for these sites is equal to 0. All other sites were used to calculate the pairwise genetic distance using the L1-norm. Data and code are located at https://github.com/GhedinSGS/TCD_Mice.

### Quantification and statistical analysis

All experiments in mice were repeated at least twice, with representative results from one experiment shown. Statistical analysis was performed in GraphPad Prism 10.1.1 (Prism, La Jolla, CA, USA) using Student’s *t* test to compare two groups and unpaired one-way analysis of variance (ANOVA) to compare more than two groups. Statistical significance was defined as *P* values less than 0.05.

Antibody neutralization was quantified by determining the foci count for each sample done in duplicates with the aid of the Viridot program (*89*). To calculate the neutralization titers, the following formula was applied: 1 − (mean number of foci in the presence of sera divided by foci at the highest dilution of the corresponding sera sample). The FRNT_50_ titers were estimated through four-parameter nonlinear regression in GraphPad Prism 8.4.3. Samples that did not exhibit neutralization at 50% were plotted at 20 and used for calculating the geometric mean. qRT-PCR results are expressed relative to mouse GAPDH expression for the same sample and were calculated using the ΔΔCT relative quantitation method as compared to mock age-matched controls.
