# Supplementary material for: CD4+ and CD8+ T cells are required to prevent SARS-CoV-2 persistence in the nasal compartment
Source: Sci Adv. 2024 Aug 23;10(34):eadp2636. doi: 10.1126/sciadv.adp2636 (PMC11343035; doi:10.1126/sciadv.adp2636)
Supplement: Supplementary file 1 — Figs. S1 to S8 [file sciadv.adp2636_sm.pdf]

Science Advances
AAAS

Supplementary Materials for

CD4$^+$ and CD8$^+$ T cells are required to prevent SARS-CoV-2 persistence in the nasal compartment

Meenakshi Kar *et al.*

Corresponding author: Mehul S. Suthar, mehul.s.suthar@emory.edu

Sci. Adv. **10**, eadp2636 (2024)
DOI: 10.1126/sciadv.adp2636

This PDF file includes:

Figs. S1 to S8

SUPPLEMENTARY FIGURES

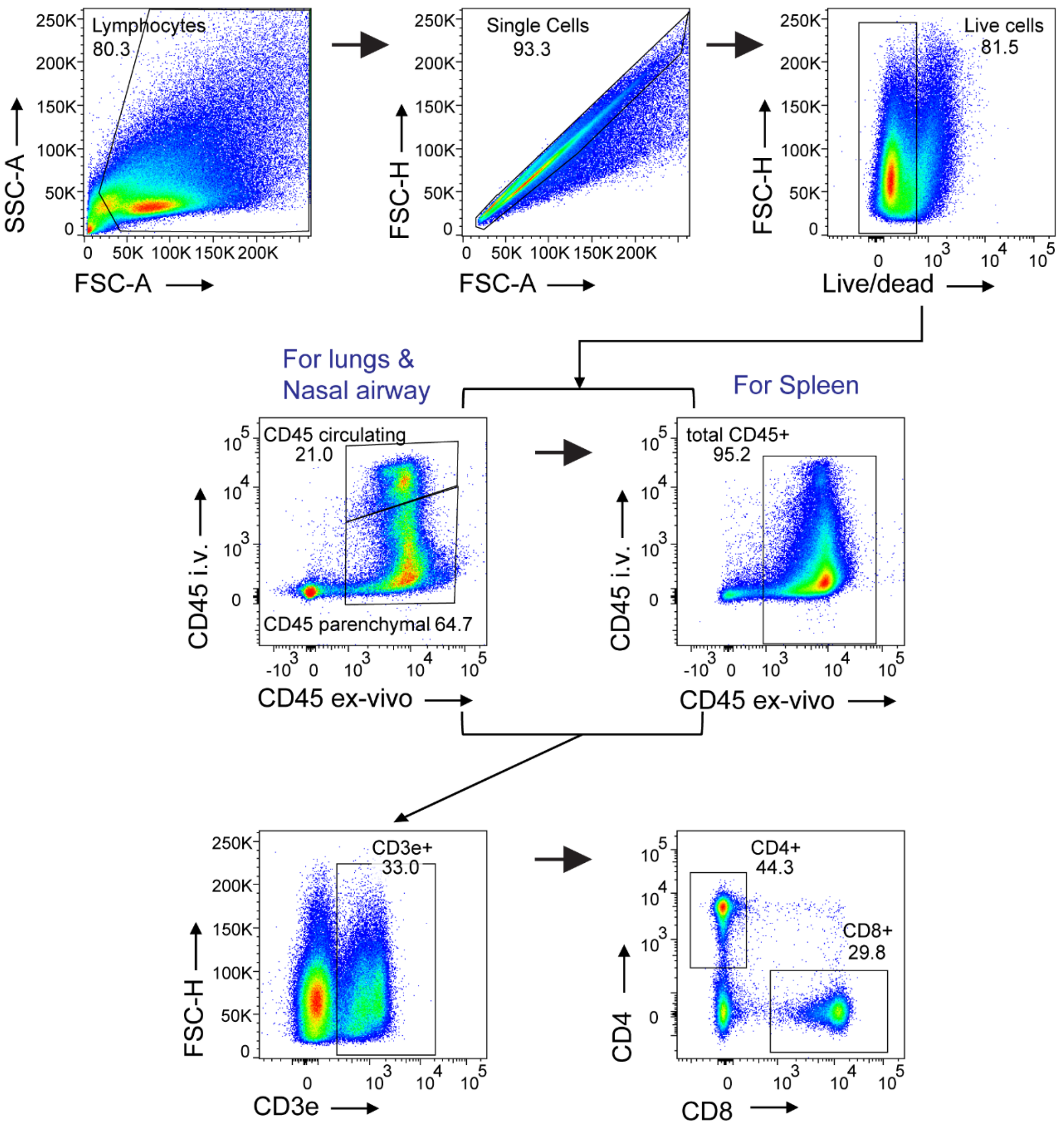

Supplementary Figure S1- Gating strategy for flow cytometry analysis of CD4+ and CD8+ T cells at day 8 pi. C57Bl/6 mice were infected with SARS-CoV-2 B.1.351 and spleen, lungs and nasal airway tissues were harvested at day 7 pi. Five minutes prior to euthanasia, mice were intravitally labelled with CD45:PE (injected via the retro-orbital route). Tissues were processed to a single-cell suspension and analyzed via flow cytometry. Total cell populations were gated on (FSC-SSC), then singlets, then on Live (Ghost Dye 780). Lung infiltrating cells were identified as CD45 IV- and CD45 ex-vivo+. Tissue parenchymal

lymphocytes in case of lungs and nasal airways and total CD45+ cells in case of spleen were then gated for CD3+ cells which were further gated for CD4 and CD8+ T cells.

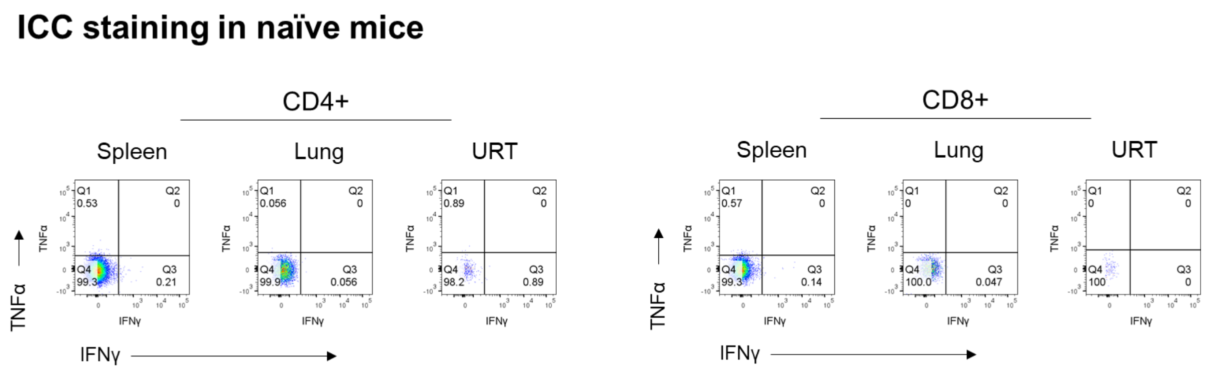

Supplementary Figure S2- Intracellular cytokine staining in naïve mice. Naïve mice were stimulated with peptides and intracellular cytokine staining performed as described in the materials and methods.

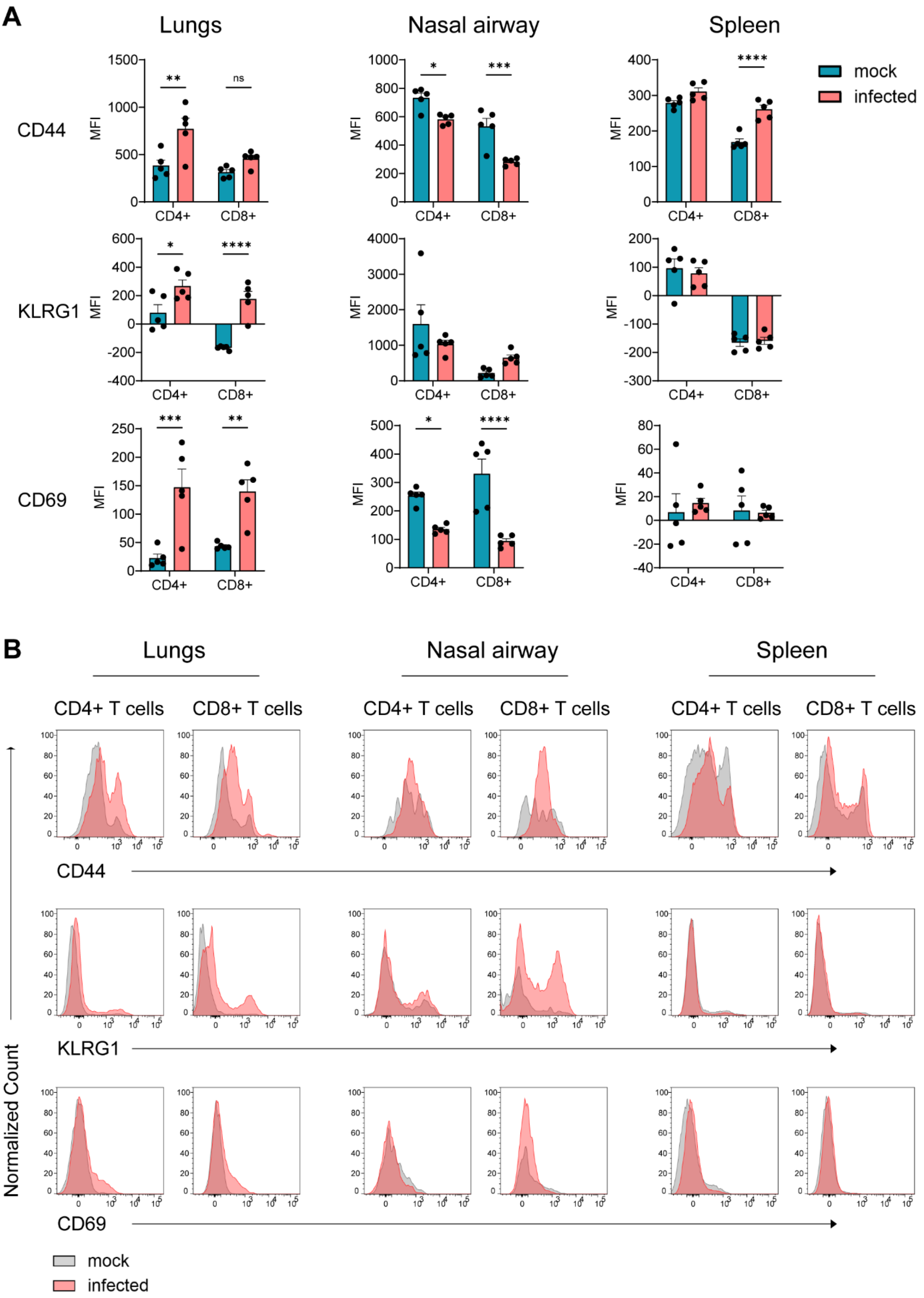
A
Lungs
Nasal airway
Spleen
mock
infected
CD44
KLRG1
CD69
MFI
CD4+
CD8+
B
CD4+ T cells
CD8+ T cells
Normalized Count

Supplementary Figure S3- Activation markers in CD4+ and CD8+ T cells. Lungs, URT and spleen tissues were harvested from C57Bl/6 mice infected with SARS-CoV-2 B.1.351 at day 7 pi and analysed for activation markers CD44, KLRG1 and CD69 by flow cytometry. (A) Mean Fluorescence Intensity (MFI) (A) and representative histogram overlay plots (B) for indicated markers in CD4+ and CD8+ T cells in infected samples compared to mock in lungs, nasal airways, and spleen at day 7 pi.

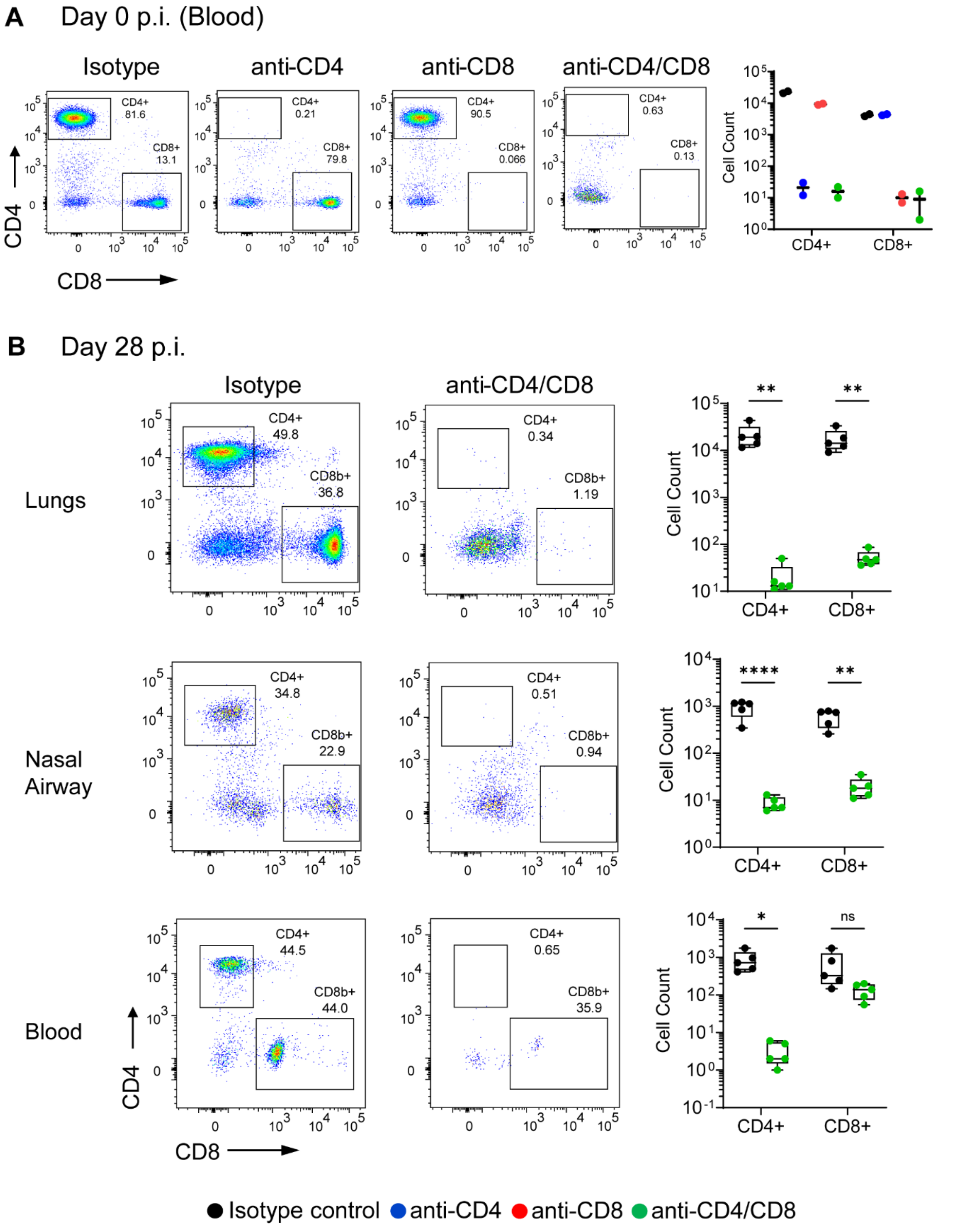

Supplementary Figure S4- CD4+ and CD8+ T cells depletion assessment at day 0 and day 28 pi in lungs and nasal airways. (A) Whole blood from mice depleted for either CD4+ T cells or CD8+ T cells or both were subjected to CD4+ and CD8+ staining to assess depletion

efficiency as compared to the isotype control at day 0 pi. **(B)** Cells isolated from lungs, nasal airways, and whole blood at day 28 pi, were assessed for depletion efficiency as compared to the isotype control. The analysis was done on CD3+ T cells which were gated on live and single CD45+ lymphocytes. Cell counts depicted as insets against the respective time point and tissue type.

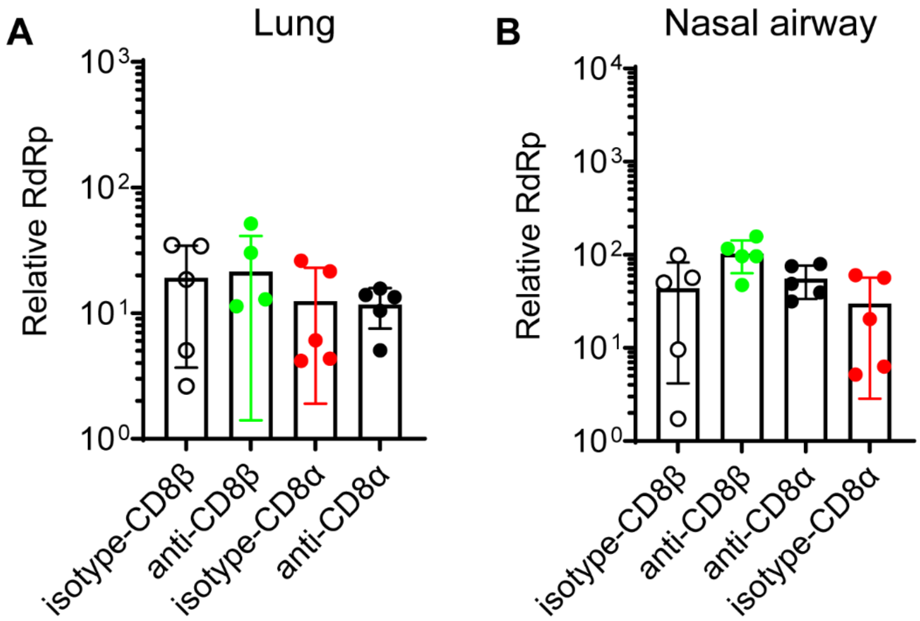

Supplementary Figure S5- comparison of effect of CD8+T cell depletion using CD8β or CD8α antibody on viral RNA levels in lungs and nasal airways at day 28 pi Mice were either depleted using CD8β or CD8α antibody and assessed for viral RdRp levels in **(A)** lungs and **(B)** nasal airways at day 28 pi by qRT-PCR. RNA levels were compared with individual isotype controls. n=5 mice for each group.

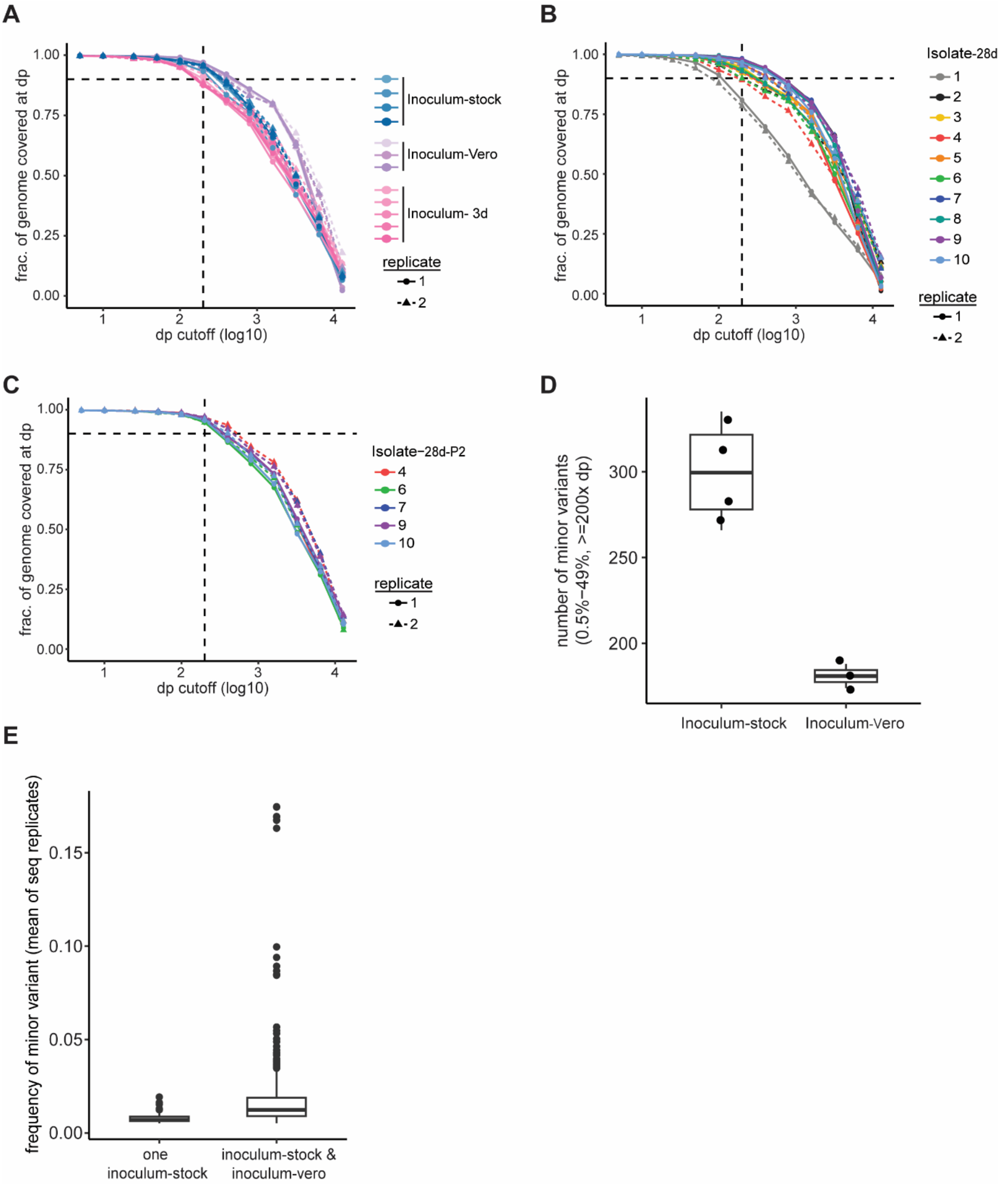

Supplementary Figure S6- Quality checks for SARS-CoV-2 sequencing data. **(A-C)** Fraction of the genome covered (y-axis) across different read depths (x-axis) for (A) the inoculum stock, inoculum-Vero, and inoculum-C57BL/6 controls, **(B)** day 28 pi CD4+/CD8+ tandem T-cell depleted mouse samples, and **(C)** the 5 mouse isolates passaged once in VeroE6-TMPRSS2 cells. Data for each sequencing replicate are plotted and differentiated by the point

shape and line type. **(D)** The number of minor variants found at 0.5-49% in the inoculum stock and inoculum-Vero controls. **(E)** The frequency of minor variants (y-axis) found in a single stock sample versus the frequency of minor variants present in at least one inoculum stock and one inoculum-Vero sample (x-axis).

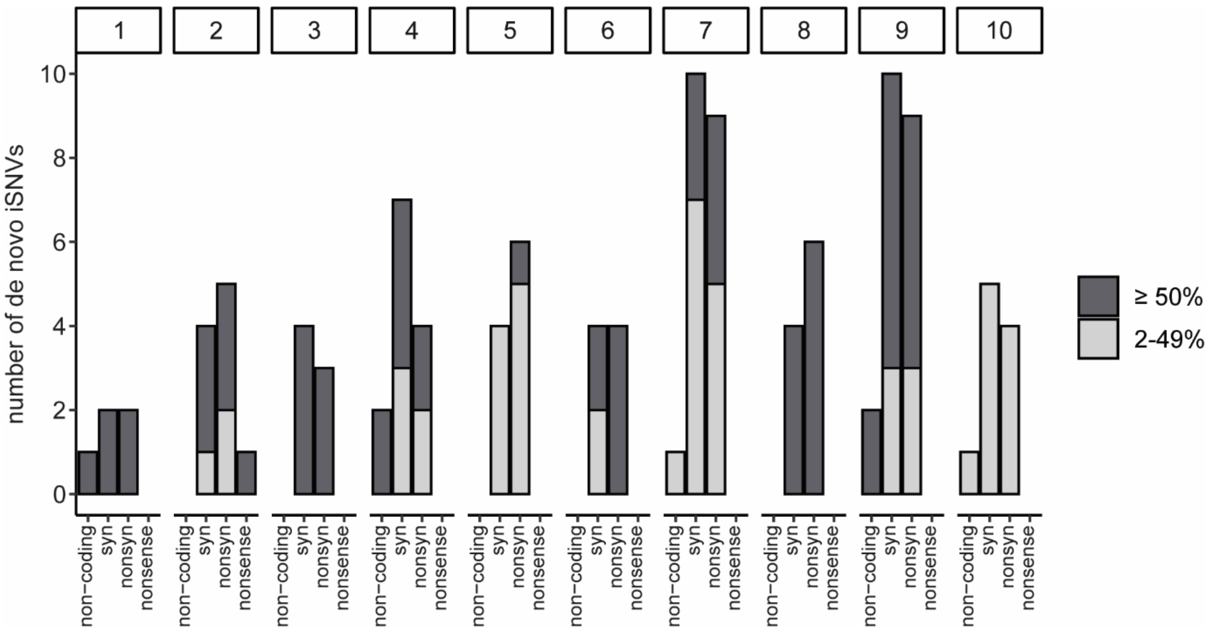

Supplementary Figure S7- iSNV richness for each mouse isolate. The number of high-frequency (dark gray) and low-frequency (light gray) *de novo* iSNVs (y-axis) in each mouse isolate separated by mutation type (non-coding, synonymous "syn", nonsynonymous "nonsyn", and nonsense).

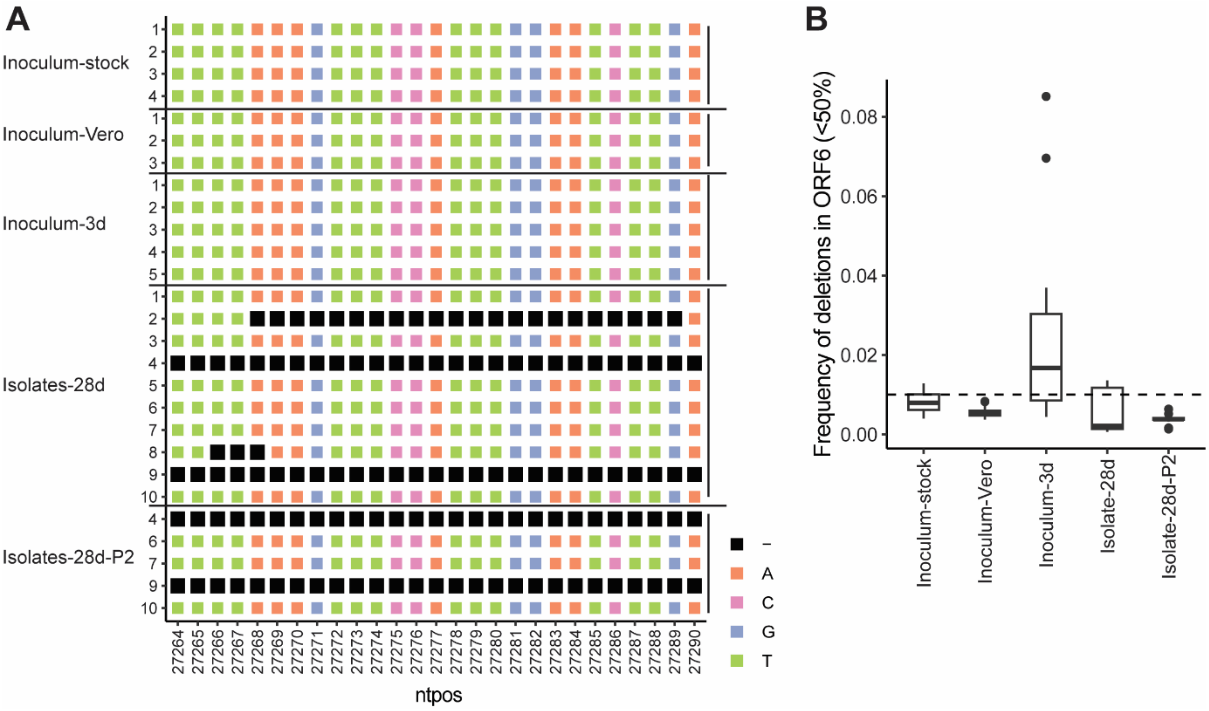

Supplementary Figure S8- Consensus level deletions in mouse isolates. (A) ORF6 consensus sequences from nucleotide positions 27264-27290 across all inoculum and mouse isolate samples. Box colors represent the nucleotide found at each position. Black squares represent positions identified as a deletion. **(B)** The relative frequency of ORF6 27264-27290 deletions in other samples where it did not reach consensus levels.
